# Supplementary material for: Grainyhead 1 acts as a drug-inducible conserved transcriptional regulator linked to insulin signaling and lifespan
Source: Nat Commun. 2022 Jan 10;13:107. doi: 10.1038/s41467-021-27732-4 (PMC8748497; doi:10.1038/s41467-021-27732-4)
Supplement: Supplementary file 1 — Supplementary Information [file 41467_2021_27732_MOESM1_ESM.pdf]

SUPPLEMENTARY INFORMATION

Grainyhead 1 acts as a drug-inducible conserved transcriptional regulator linked to insulin signaling and lifespan

Giovanna Grigolon, Elisa Araldi, Reto Erni, Jia Yee Wu, Carolin Thomas, Marco La Fortezza, Beate Laube, Doris Pöhlmann, Markus Stoffel, Kim Zarse, Erick M. Carreira, Michael Ristow, Fabian Fischer

Corresponding author: Michael Ristow (email: michael-ristow@ethz.ch)

This PDF file includes:

SUPPLEMENTARY FIGURES 1 to 5 Pages 2 – 8
SUPPLEMENTARY TABLES 1 to 3 Pages 9 – 11
SUPPLEMENTARY METHODS Pages 12 – 23
SUPPLEMENTARY REFERENCES Page 24

SUPPLEMENTARY FIGURES

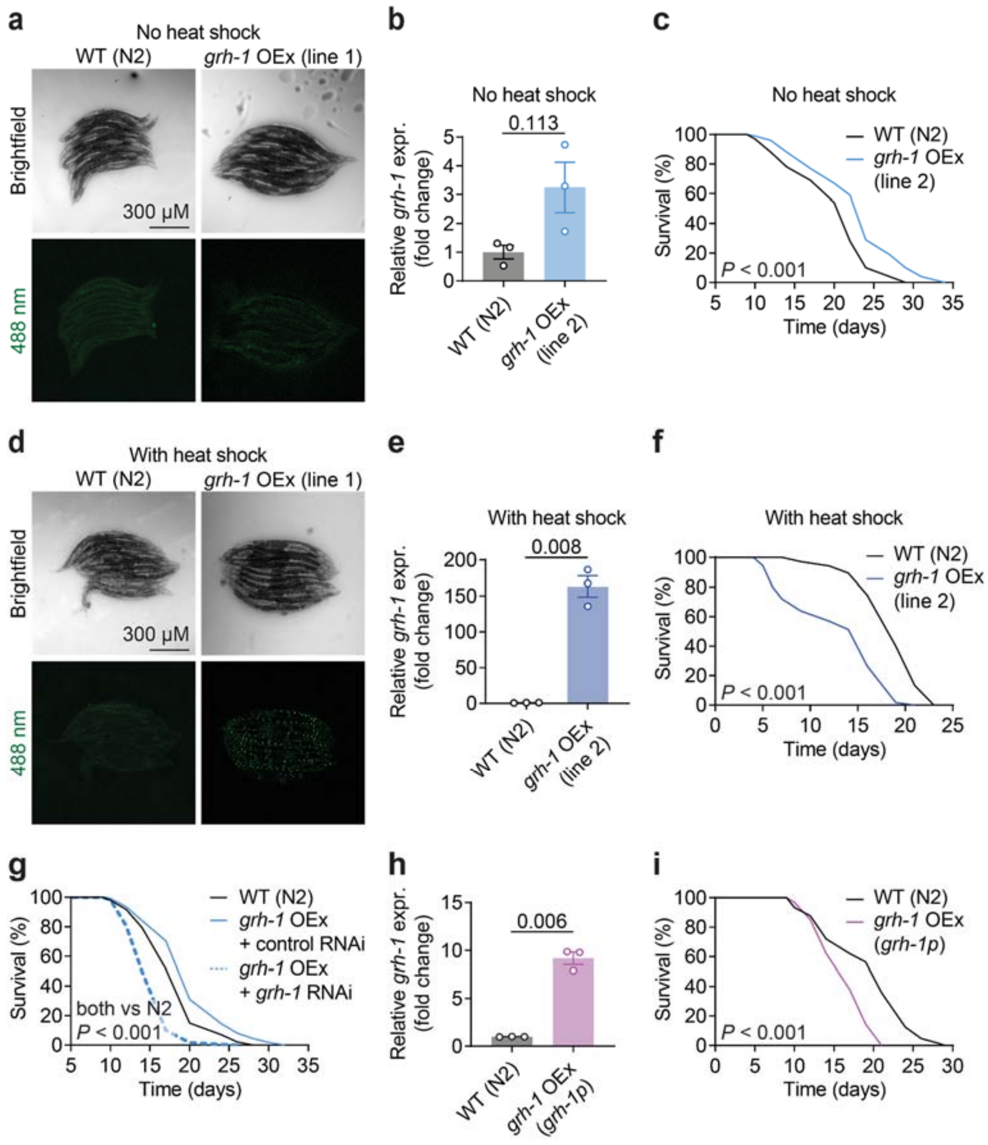

Supplementary Figure 1. Nematodal *grh-1* impacts aging phenotypes.

a Individual microscopy images related to Fig. 1d. b RT-qPCR of *grh-1* in WT (N2) vs. *grh-1* OEx (line 2) without heat shock (n = 3 for both). **c** Lifespan assay of WT (N2) vs. *grh-1* OEx (line 2) on OP50 bacteria without heat shock. **d** Individual microscopy images related to Fig. 1g. **e, f** As in (**b, c**) with heat shock (RT-qPCR with n = 3 for both). **g** Lifespan assay of *grh-1* OEx with *grh-1* RNAi compared to control RNAi and WT (N2). **h** RT-qPCR of *grh-1* in WT (N2) vs. a line overexpressing *grh-1* under control of its endogenous promoter (*grh-1p*; n = 3 for both). **i** Lifespan assay of WT (N2) vs. the same *grh-1* overexpressing line as in (**h**). Data in bar graphs are mean ± SEM, with sample sizes as stated and individual data points representing biological replicates. *P*-values were determined with two-tailed unequal variances *t*-tests of the indicated comparisons of unpaired control vs. treatment groups. *P*-values of *C. elegans* lifespan assays were determined by log-rank test. See Supplementary Data 1 for detailed lifespan assay statistics. Source data are provided as a Source Data file.

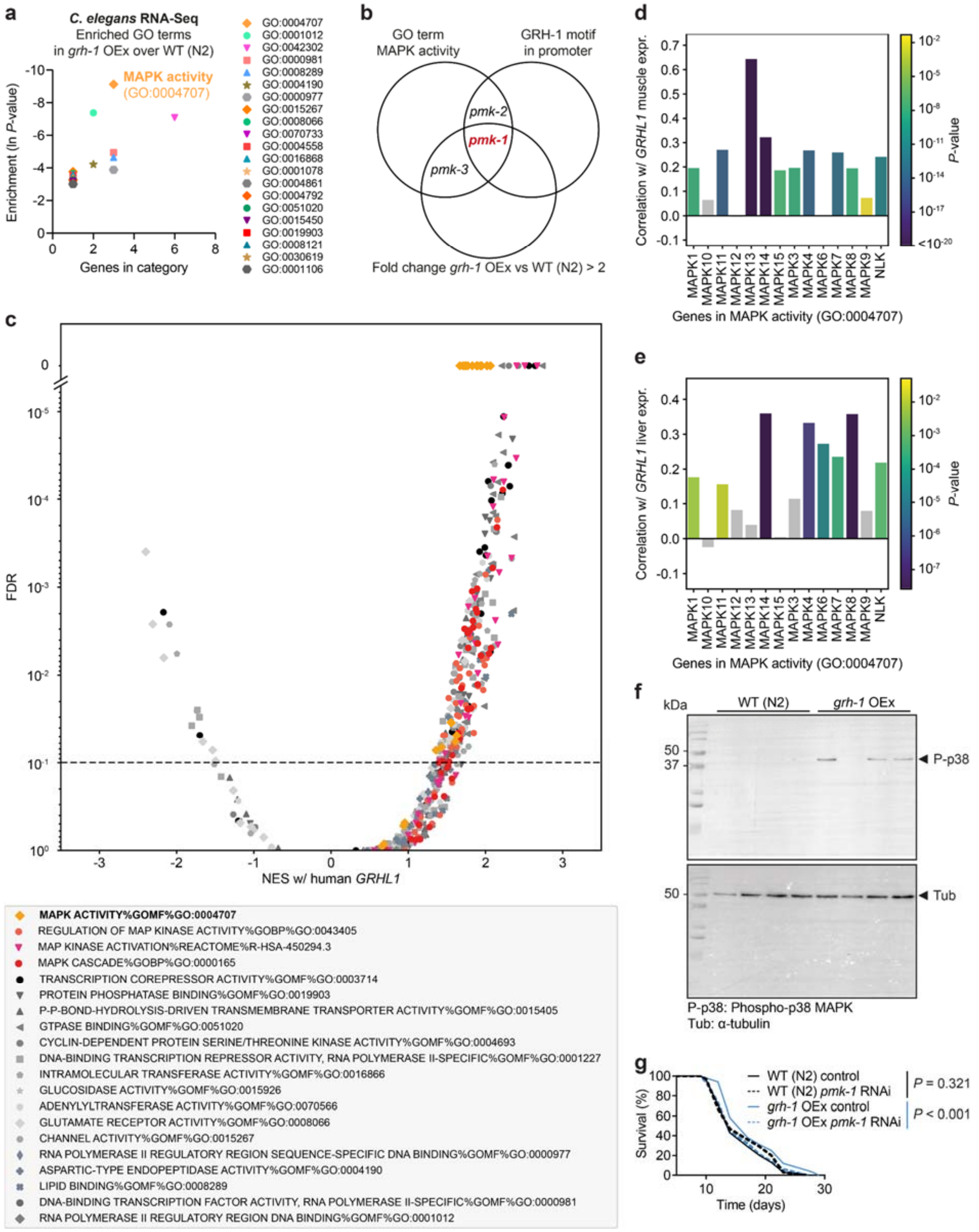

Supplementary Figure 2. Nematodal *grh-1* and human *GRHL1* are linked to MAP kinase activity.

a Plot of enriched GO terms among genes upregulated in *grh-1* OEx vs. WT, as determined by RNA-Seq. See Supplementary Data 2. **b** Venn intersection of *C. elegans* 'MAPK activity' (GO:0004707) genes that have GRH-1 binding motifs in their promoter and are overexpressed at least 2-fold in *grh-1* OEx. See Supplementary Data 2 and 3. **c** Normalized enrichment score (NES) and FDR of gene set enrichment analysis of genes correlating with human *GRHL1* in non-gender-specific tissues. Only gene sets also enriched in *grh-1* OEx RNA-Seq are shown. **d, e** Pearson's r correlation coefficients and two-tailed test *P*-values between GRHL1 and genes in GO term 'MAPK activity' (GO:0004707) in human muscle (**d**) or liver (**e**) samples, derived from the GTEx database. Gray bars represent non-significant correlations (*P*-value > 0.05). **f** Full western blot image related to Fig. 1k (WT and *grh-1* OEx nematodes *n* =

4 independent samples each). **g** Lifespan assay of WT (N2) and *grh-1* OEx on post-developmental *pmk-1* RNAi compared to control vector L4440. *P*-values of *C*. *elegans* lifespan assays were determined by log-rank test. See Supplementary Data 1 for detailed lifespan assay statistics. Source data are provided as a Source Data file.

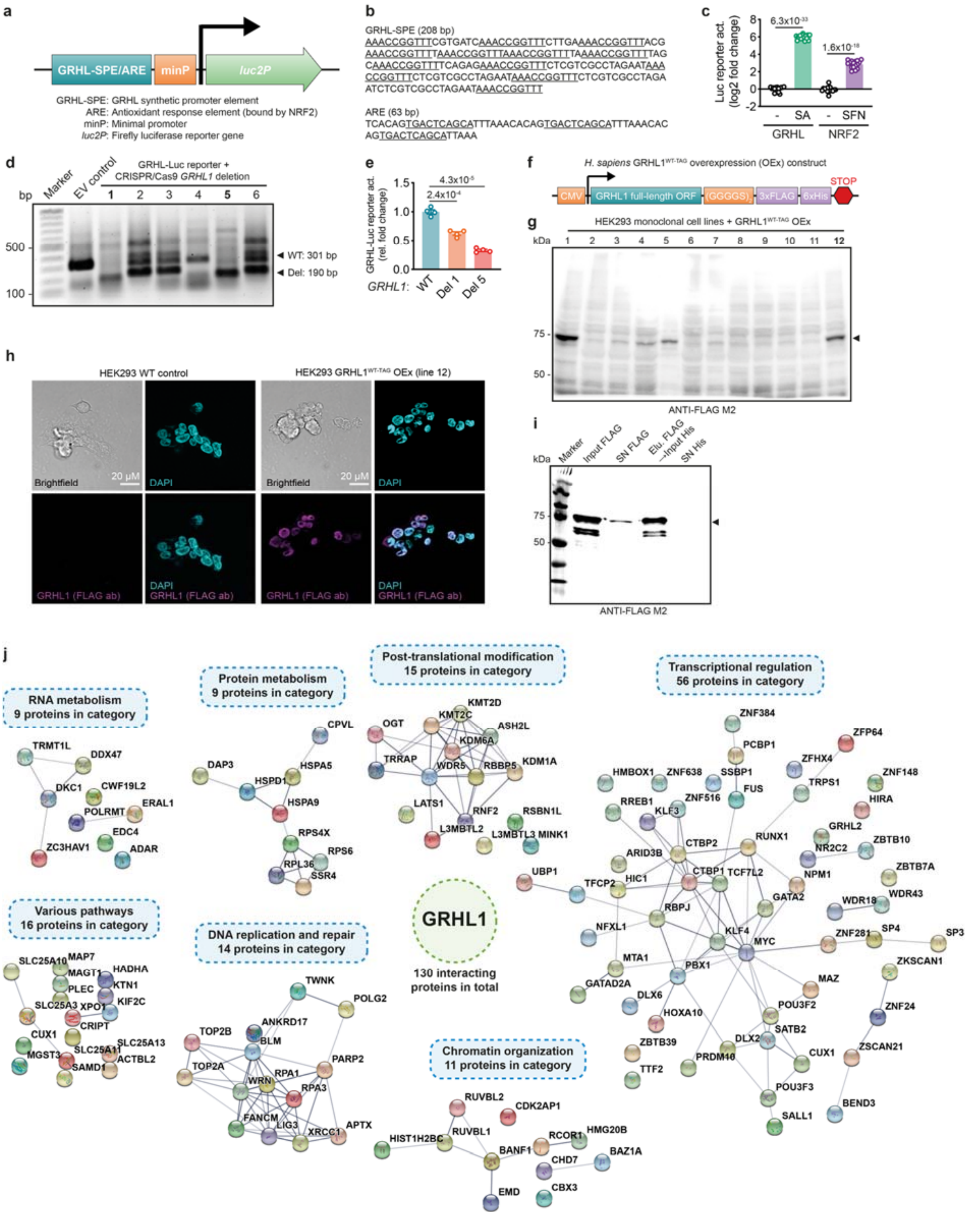

Supplementary Figure 3. Human GRHL and NRF2 reporters and human GRHL1 protein interactome.

a Scheme of luciferase reporter constructs for GRHL and NRF2. **b** Nucleotide sequences of the GRHL-synthetic promoter element (SPE) and the NRF2 antioxidant response element (ARE). Binding motifs underlined. **c** Activation of stable GRHL and NRF2 luciferase reporter cell lines with control compounds Scriptaid (SA) and sulforaphane (SFN) as log2 fold change over DMSO control (*n* = 16 for all). **d** Genotyping PCR for constitutive CRISPR/Cas9-mediated deletion of *GRHL1* in GRHL luciferase reporter clones 1 and 5 (in bold). Size of wild-type (WT) and deletion (Del) fragment as indicated. **e** Luciferase assay of GRHL luciferase reporter with wild-type (WT) *GRHL1* or deletion thereof (*n* = 4 for all). **f** Scheme of 3xFLAG-6xHis tagged wild-type human GRHL1 ($GRHL1^{WT\text{-}TAG}$) overexpression construct. **g** Western blot of HEK293 monoclonal cell lines stably transfected with the construct in (**f**) and detection of 74.5 kDa $GRHL1^{WT\text{-}TAG}$ with

FLAG antibody. Line 12 (in bold) was used for subsequent experiments. **h** Microscopy images of a HEK293 wild-type (WT, left) cell line and the GRHL1^{WT-TAG}-overexpressing line 12 (right), with brightfield, DAPI staining, GRHL1 immunofluorescence (using the same FLAG antibody as in **a**), and overlay of the latter two, showing GRHL1^{WT-TAG} nuclear localization. **i** Western blot of representative GRHL1^{WT-TAG} TAP fractions. The FLAG elution fraction was used as input for the His purification procedure, and His beads with bound proteins were used as input for MS/MS. Successful GRHL1^{WT-TAG} purification verified for all applicable samples (n = 3 each) used in MS/MS. **j** Clustered protein-protein interaction network of all proteins here identified as GRHL1 interactors, with each of the 130 proteins assigned to one of seven categories according to their manually curated functional classification. Edges represent known protein-protein associations, with darker color indicating stronger data support. Visualized with the help of STRING (https://string-db.org). The interaction network of proteins classified as impacting post-translational modifications (at the top) is also depicted in Fig. 4e. See Supplementary Data 5. Data in bar graphs are mean ± SEM, with sample sizes as stated and individual data points representing biological replicates. P-values were determined with two-tailed unequal variances t-tests of the indicated comparisons of unpaired control vs. treatment groups. Source data are provided as a Source Data file.

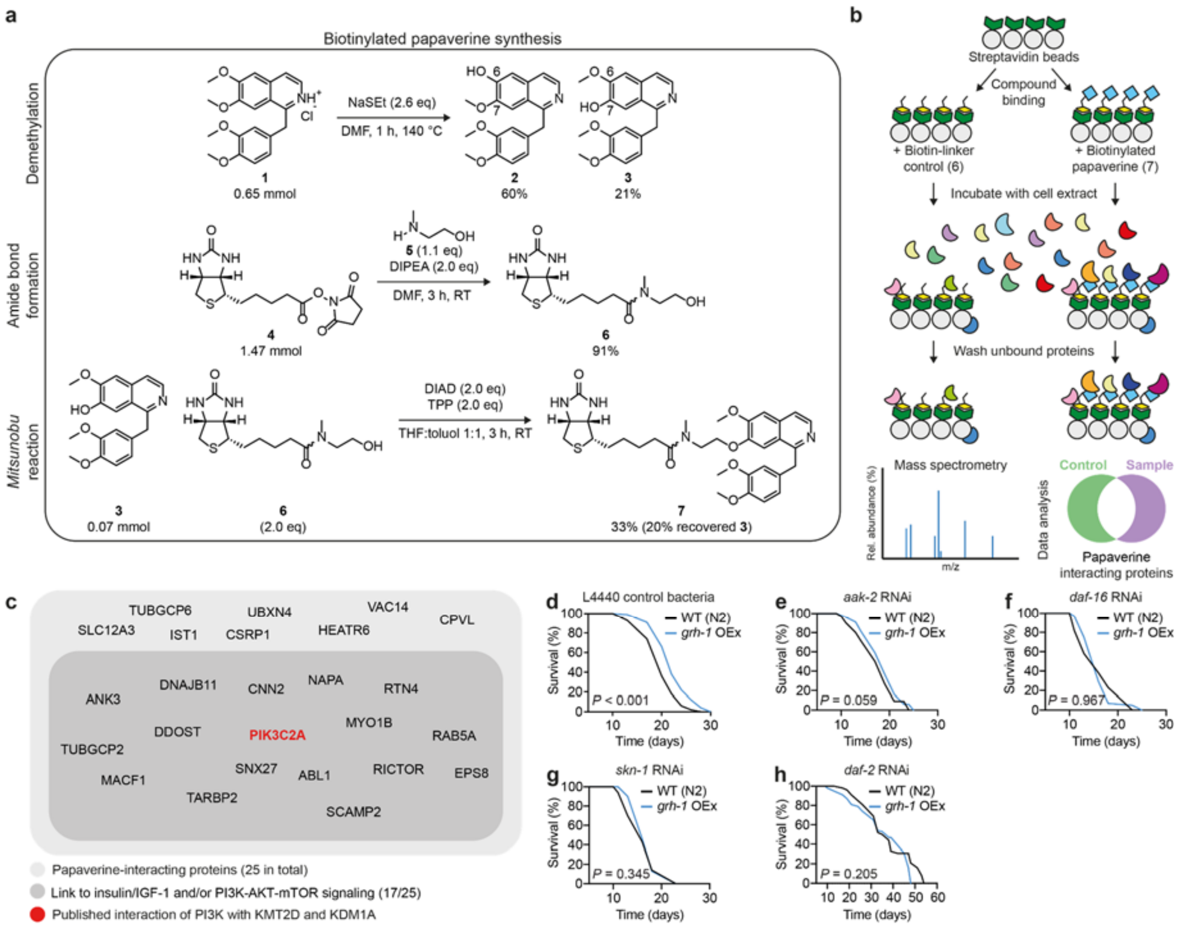

Supplementary Figure 4. Protein pull-down using biotinylated papaverine and *C. elegans* IIS-related lifespan assays.

a Synthesis scheme of biotinylated papaverine (7) from papaverine hydrochloride (1). See Supplementary Methods for details. **b** Experimental design of pull-down experiment using biotin-linker control (compound 6 in **a**) and biotinylated papaverine (compound 7 in **a**) coupled to streptavidin beads and incubated with HEK293 total cell extract. **c** Overview of biotinylated papaverine-interacting proteins identified following (**b**), highlighting their relation to insulin/IGF-1 and/or PI3K-AKT-mTOR signaling and indicating published interactions with KMT2D and KDM1A. See Supplementary Table 2. **d-h** Lifespan assays using WT (N2) nematodes and *grh-1* OEx, treated post-developmentally with the control vector L4440 (**d**) or individual RNAis (**e-h**) as indicated. *P*-values of *C. elegans* lifespan assays were determined by log-rank test. See Supplementary Data 1 for detailed lifespan assay statistics. Source data are provided as a Source Data file.

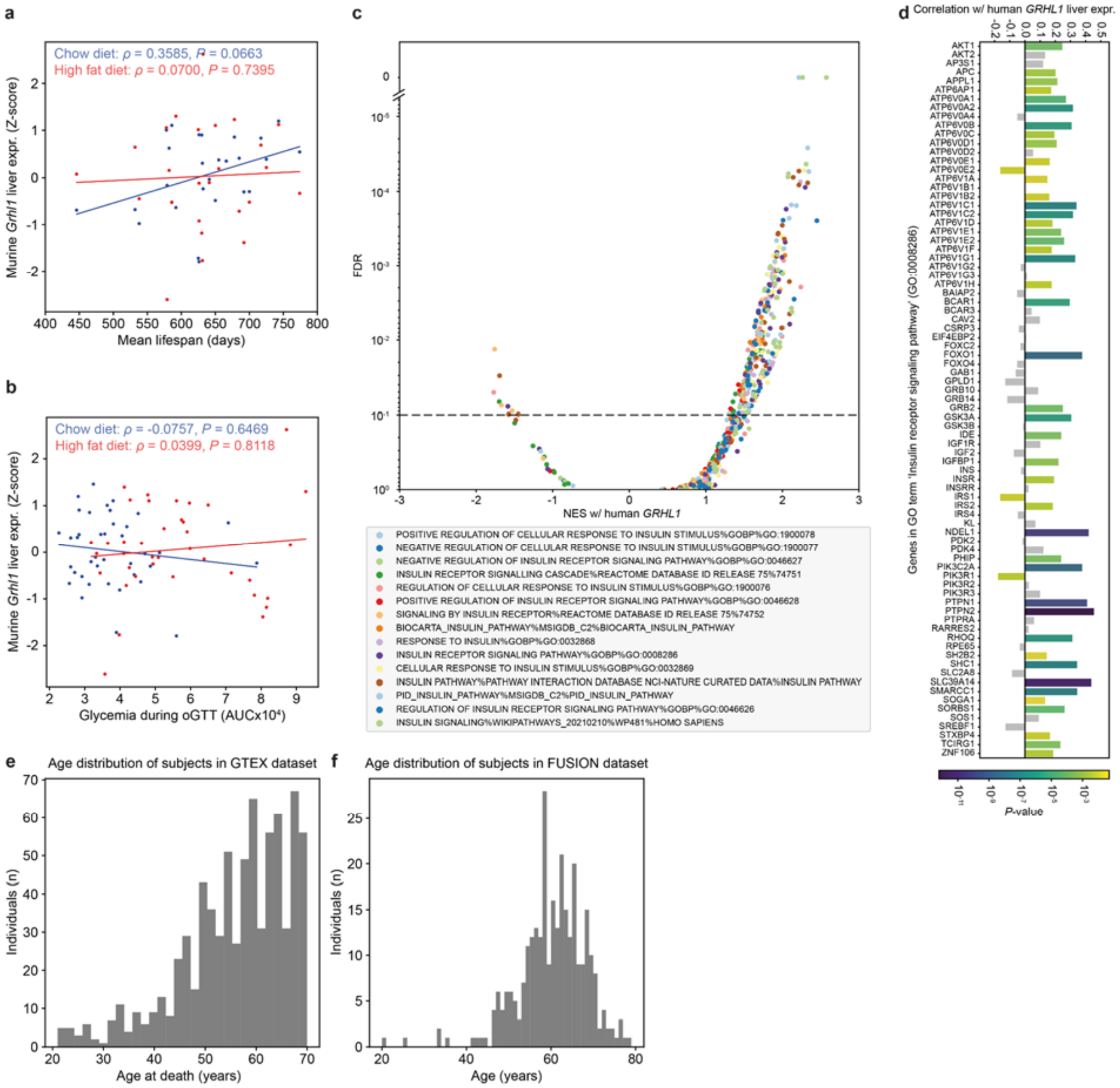

Supplementary Figure 5. Correlations of human *GRHL1* and murine *Grhl1* with insulin signaling and metabolic parameters.

a Correlation by Spearman's rho and two-tailed test *P*-values between *Grhl1* expression in liver (Z-score normalized) and mean lifespan of chow and high fat diet fed mice from the BXD mouse genetic reference population. **b** Similar as in **a**, depicting correlation of *Grhl1* expression in liver with area under the curve (AUC) glycemia during oral glucose tolerance test (oGTT). **c** Normalized enrichment score (NES) and FDR of gene sets enrichment analysis of genes correlating with human *GRHL1* in non-gender-specific tissue. Only gene sets related to insulin signaling are shown. **d** Pearson's r correlation coefficients and two-tailed test *P*-values between *GRHL1* and genes in GO term 'Insulin receptor signaling pathway' (GO: 0008286) in human liver samples, derived from the GTEx database. Gray bars represent non-significant correlations (*P*-value > 0.05). **e** Age distribution of subjects in GTEx data set who died of so-called 'accidental' deaths and included in the analysis. **f** Age distribution of subjects in the FUSION data set.

SUPPLEMENTARY TABLES

Supplementary Table 1. Post-translational modifications identified for GRHL1$^{WT\text{-}TAG}$.

Overview of post-translational modifications of GRHL1$^{WT\text{-}TAG}$, as determined by mass spectrometry of the recombinant protein after tandem-affinity purification from nuclei of HEK293 cells treated with DMSO or papaverine. Listed are the modified amino acid, its position in GRHL1$^{WT\text{-}TAG}$, the type of modification, and in how many samples X/3 DMSO and/or papaverine the particular modification was identified. The data are based on the peptide identification results obtained using PEAKS X search engine and stringent filters.

Amino Acid	Position	Modification	DMSO	Papaverine
Lys (K)	8	Acetylation	3	3
Arg (R)	9	Dimethylation	0	1
Met (M)	48	Oxidation	3	3
Met (M)	49	Oxidation	3	3
Ser (S)	95	Phosphorylation	0	1
Lys (K)	116	Dimethylation	0	2
Lys (K)	134	Acetylation	1	1
Thr (T)	139	Phosphorylation	0	1
Met (M)	152	Oxidation	3	3
Thr (T)	208	Phosphorylation	3	3
Met (M)	234	Oxidation	2	3
Met (M)	237	Oxidation	2	3
Met (M)	271	Oxidation	1	2
Met (M)	305	Oxidation	3	3
Lys (K)	370	Acetylation	0	1
Ser (S)	374	Phosphorylation	0	1
Ser (S)	442	Phosphorylation	2	2
Met (M)	455	Oxidation	3	3
Ser (S)	493	Phosphorylation	1	1
Ser (S)	539	Phosphorylation	1	1
Met (M)	547	Oxidation	3	3
Met (M)	557	Oxidation	3	3
Met (M)	586	Oxidation	3	3
Lys (K)	592	Ubiquitylation	0	1

Supplementary Table 2. Proteins co-purifying with biotinylated papaverine.

Overview of proteins co-purifying with biotinylated papaverine, as determined by mass spectrometry of samples resulting from a protein pull-down assay using the tagged compound mixed with HEK293 total cell extracts. Listed for each papaverine-interacting protein are its corresponding gene name, Swiss-Prot ID, and label-free quantification (LFQ) and unique peptide (UP) fold change (fc) over the biotinylated linker control. The last column additionally lists PubMed IDs (PMIDs) or digital object identifiers (DOI) of publications indicating a link of the respective protein to insulin signaling and/or the PI3K-AKT-mTOR signaling axis.

Gene name	Swiss-Prot ID	LFQ fc	UP fc	PMIDs/DOI
ABL1	P00519	4.9	5.0	32393599
ANK3	Q12955	4.4	5.5	16377635
CNN2	Q99439	3.3	3.0	28915602
CPVL	Q9H3G5	4.0	3.0	NA
CSRP1	P21291	4.9	3.0	NA
DDOST	P39656	3.9	6.0	29802324, 31541173
DNAJB11	Q9UBS4	3.5	4.0	https://doi.org/10.1101/699132
EPS8	Q12929	5.8	9.0	20209148
HEATR6	Q6AI08	4.2	3.0	NA
IST1	P53990	5.2	3.0	NA
MACF1	Q9UPN3	14.1	9.7	31260729
MYO1B	O43795	15.2	5.0	31349190
NAPA	P54920	8.5	5.0	10381355
PIK3C2A	O00443	3.2	3.3	26489763, 30567315, 31428587
RAB5A	P20339	3.3	6.0	23434372, 26100075
RICTOR	Q6R327	3.4	3.0	15718470, 17967879
RTN4	Q9NQC3	3.2	3.0	30078441
SCAMP2	O15127	4.8	5.0	8360193
SLC12A3	P55017	3.2	4.0	NA
SNX27	Q96L92	12.7	4.0	29117568, 29284659
TARBP2	Q15633	3.7	3.0	25843719
TUBGCP2	Q9BSJ2	5.1	9.0	33458610
TUBGCP6	Q96RT7	4.3	4.3	NA
UBXN4	Q92575	3.6	4.0	NA
VAC14	Q08AM6	6.0	3.0	NA

Supplementary Table 3. Nucleotide sequences.

Full sequences of nucleotides related to RT-qPCR experiments, vector cloning, vector mutagenesis, and genotyping.

Nucleotide name	Related gene	Organism	Use	Sequence (5'-3')
grh-1_qPCR_fwd	*grh-1*/Y48G8AR.1	*C. elegans*	RT-qPCR	ACGAGCAAATTTACCAGCCG
grh-1_qPCR_rev	*grh-1*/Y48G8AR.1	*C. elegans*	RT-qPCR	GGACATCTTATCCGGGTCGG
Y45F10D.4_qPCR_fwd	*iscu-1*/Y45F10D.4	*C. elegans*	RT-qPCR	GTCGCTTCAAATCAGTTCAGC
Y45F10D.4_qPCR_rev	*iscu-1*/Y45F10D.4	*C. elegans*	RT-qPCR	GTTCTTGTCAAGTGATCCGACA
attB1_grh-1_fwd	*grh-1*/Y48G8AR.1	*C. elegans*	Cloning	GGGGACAAGTTTGTACAAAAAAGCAGGCTATGTCATTCCAACTTGACCAATCA
attB2_grh-1_rev	*grh-1*/Y48G8AR.1	*C. elegans*	Cloning	GGGGACCACTTTGTACAAGAAAGCTGGGCATGAGTTGGAATACGAGTTTGGA
attB4_hsp16.2p_fwd	*hsp-16.2*/Y46H3A.3	*C. elegans*	Cloning	GGGGACAACTTTGTATAGAAAAGTTGATTTTCGAAGTTTTTTAGAT
attb1R_hsp16.2p_rev	*hsp-16.2*/Y46H3A.3	*C. elegans*	Cloning	GGGGACTGCTTTTTTGTACAAACTTGGATTATAGTTTGAAGA
attB4_grh-1p_fwd	*grh-1*/Y48G8AR.1	*C. elegans*	Cloning	GGGGACAACTTTGTATAGAAAAGTTGGCGAAATTTCCTTCAATGCA
attB1R_grh-1p_rev	*grh-1*/Y48G8AR.1	*C. elegans*	Cloning	GGGGACTGCTTTTTTGTACAAACTTGTCTGGAATGATAATTAATGATATATT
GRHL1_NheI_fwd	*GRHL1*	*H. sapiens*	Cloning	ATCGCTAGCATGACACAGGAGTACGACAAC
GRHL1_KpnI_rev	*GRHL1*	*H. sapiens*	Cloning	ATGGTACCGATCTCCGTCAGGGTGAGC
KpnI_L3F6H_BamHI	None	None	Cloning	GGTACCGGCGGCGGCGGCGGCAGCGGCGGCGGCGGCAGCGACTACAAGGACCA<br>CGACGGCGACTACAAGGACCACGACATCGACTACAAGGACGACGACGACAAGC<br>ACCACCACCACCACCACTAAGGATCC
KpnI_GRHL-SPE_NheI	*GRHL1-3*	*H. sapiens*	Cloning	GGTACCAAACCGGTTTCGTGATCAAACCGGTTTCTTGAAAACCGGTTTACGAA<br>ACCGGTTTTAAACCGGTTTAAACCGGTTTTAAAACCGGTTTTAGCAAACCGGT<br>TTTCAGAGAAACCGGTTTCTCGTCGCCTAGAATAAACCGGTTTCTCGTCGCCT<br>AGAATAAACCGGTTTCTCGTCGCCTAGAATCTCGTCGCCTAGAATAAACCGGT<br>TTGAGCTCGCTAGC
KpnI_ARE_NheI	*NRF2*	*H. sapiens*	Cloning	GGTACCTCACAGTGACTCAGCATTTAAACACAGTGACTCAGCATTTAAACACA<br>GTGACTCAGCATTAAAGAGCTCGCTAGC
GRHL1_exon5_sg_top	*GRHL1*	*H. sapiens*	Cloning	CACCGTCCGCAGACTGAGATCCGAG
GRHL1_exon5_sg_btm	*GRHL1*	*H. sapiens*	Cloning	AAACCTCGGATCTCAGTCTGCGGAC
GRHL1_exon8_sg_top	*GRHL1*	*H. sapiens*	Cloning	CACCGCAACACTATCAGTAACATCG
GRHL1_exon8_sg_btm	*GRHL1*	*H. sapiens*	Cloning	AAACCGATGTTACTGATAGTGTTGC
GRHL1_Q5_G9_R-A_fwd	*GRHL1*	*H. sapiens*	Mutagenesis	CGACAACAAAGCCCCAGTGTTGGTTCTTC
GRHL1_Q5_G9_rev	*GRHL1*	*H. sapiens*	Mutagenesis	TACTCCTGTGTCATGCTAG
GRHL1_Q5_G95_S-A_fwd	*GRHL1*	*H. sapiens*	Mutagenesis	CAAAAGAAACGCCATACCAATTGTGACAGAGC
GRHL1_Q5_G95_rev	*GRHL1*	*H. sapiens*	Mutagenesis	CTGTGATCTGGCTCAGGG
GRHL1_Q5_G116_K-A_fwd	*GRHL1*	*H. sapiens*	Mutagenesis	GCAAGTACTGGCCAATGTGCCATTTAAC
GRHL1_Q5_G116_rev	*GRHL1*	*H. sapiens*	Mutagenesis	ACTCTGTTTTCTCCAGCAG
GRHL1_Q5_G139_T-A_fwd	*GRHL1*	*H. sapiens*	Mutagenesis	AGGCCATCTGGCCGCTCCAGATA
GRHL1_Q5_G139_rev	*GRHL1*	*H. sapiens*	Mutagenesis	CTCTTATCAATGCCCAGC
GRHL1_Q5_G370_K-A_fwd	*GRHL1*	*H. sapiens*	Mutagenesis	CGATGAAGCAGCCGTTTTCATCTCTGTG
GRHL1_Q5_G370_rev	*GRHL1*	*H. sapiens*	Mutagenesis	TTGATGTCCCATGTGAAG
GRHL1_Q5_G374_S-A_fwd	*GRHL1*	*H. sapiens*	Mutagenesis	GGTTTTCATCGCCGTGAACTGCTTAAG
GRHL1_Q5_G374_rev	*GRHL1*	*H. sapiens*	Mutagenesis	TTTGCTTCATCGTTGATG
GRHL1_Q5_G592_K-A_fwd	*GRHL1*	*H. sapiens*	Mutagenesis	CAACATTGTGGCCCATTACTCCAATGAGGAC
GRHL1_Q5_G592_rev	*GRHL1*	*H. sapiens*	Mutagenesis	TCGTCCATGTTCACCAGG
GRHL1_del_fwd	*GRHL1*	*H. sapiens*	Genotyping	TGGAAAGGATAGTGGGTTGCT
GRHL1_del_rev	*GRHL1*	*H. sapiens*	Genotyping	CCACCCACCTTTGCTTCATC
GRHL1_wt_rev	*GRHL1*	*H. sapiens*	Genotyping	AAGATAAGGGGAAAGCCTGCT

SUPPLEMENTARY METHODS

Detailed procedures of biotinylated Papaverine synthesis

General

Unless otherwise noted, all reactions were carried out under an ambient atmosphere, and all reagents were purchased from commercial suppliers and used without further purification. Analytical thin layer chromatography (TLC) was performed on Merck silica gel 60 F254 TLC glass plates and visualized with 254 nm light and potassium permanganate or cerium ammonium molybdate solution followed by heating. Purification of reaction products was carried out by flash chromatography using Sigma-Aldrich silica gel, 60Å, 230-400 mesh under 0.3-0.5 bar pressure. ^{1}H NMR spectra were recorded on a Bruker AV-NEO 400 MHz or Bruker AV-NEO 500 MHz spectrometer and are reported in ppm with the solvent resonance employed as the internal standard ($CHCl_3$ at 7.26 ppm and DMSO-d_6 at 2.50 ppm). Peaks are reported as (s = singlet, d = doublet, t = triplet, q = quartet or m = multiplet, coupling constant(s) in Hz, integration). ^{13}C NMR spectra were recorded with ^{1}H-decoupling on a Bruker AV-NEO 101 MHz or Bruker AV-NEO 126 MHz spectrometer and are reported in ppm with the solvent resonance employed as the internal standard ($CDCl_3$ at 77.16 ppm and DMSO-d_6 at 39.52). 2D-NMR spectra (^{1}H/^{13}C HSQC, ^{1}H/^{13}C HMBC, ^{1}H COSY, ^{1}H NOESY) were recorded on a Bruker AV-NEO 500 MHz spectrometer with the solvent resonance employed as the internal standard ($CHCl_3$ at 7.26 ppm/77.16 ppm). Diagnostic cross peaks are labelled in the spectrum and interactions indicated in the chemical structure. Infrared spectra were measured neat on a Perkin-Elmer UATR Two Spectrometer. The peaks are reported as absorption maxima (n, cm^{-1}). High resolution mass spectral data were obtained at the mass spectrometry service operated by the Laboratory of Organic Chemistry at the ETHZ on a Bruker maXis – ESI-Qq-TOF-MS (ESI) and are reported as (*m/z*). Optical rotations were measured with a Jasco P-2000 Polarimeter (10 cm, 1 mL cell). Melting points were obtained on an Büchi M-560.

Synthesis and characterization of products

Synthesis of 6-desmethylpapaverine (**2**) and Pacodine (**3**):

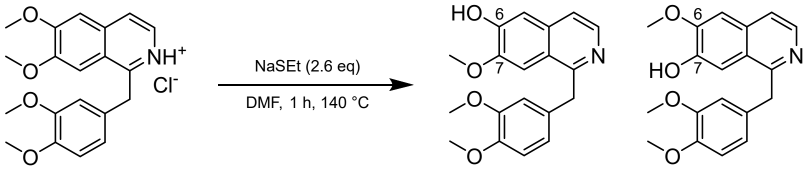

1 **2** **3**

To a solution of Papaverine hydrochloride (**1**) (250 mg, 0.65 mmol, 1.0 eq) in DMF (6.7 ml, 0.1 M) was added NaSEt (162 mg, 1.73 mmol, 2.6 eq) at RT. The reaction was stirred at 140 °C for 1 h. After allowing the reaction to reach RT, it was quenched by addition of water. The mixture was extracted three times with ethyl acetate and the combined organic layers were washed with brine, dried over $MgSO_4$, filtered and concentrated. The crude mixture was purified by flash column chromatography (SiO_2; DCM:MeOH 10:1) resulting in 6-desmethylpapaverine (**2**) as a white powder (130.0 mg, 0.40 mmol, 60.1% yield) and Pacodine (**3**) as a beige powder (45.5 mg, 0.14 mmol 21.0% yield). Reaction conditions were adapted from literature[1].

6-desmethylpapaverine (**2**):

^{1}H NMR (500 MHz, $CDCl_3$) δ = 8.31 (d, *J* = 5.7 Hz, 1H), 7.37 – 7.35 (m, 1H), 7.16 (s, 1H), 6.82 (d, *J* = 2.0 Hz, 1H), 6.81 – 6.78 (m, 1H), 6.74 (d, *J* = 8.2 Hz, 1H), 4.54 (s, 2H), 3.91 (s, 3H), 3.80 (s, 3H), 3.74 (s, 3H).

^{13}C NMR (126 MHz, $CDCl_3$) δ = 157.8, 150.0, 149.2, 148.4, 147.7, 140.3, 134.2, 132.2, 122.9, 120.6, 119.0, 112.0, 111.3, 108.9, 104.0, 56.0, 56.0, 55.9, 42.0.

IR (neat, v_{max}/cm^{-1}) 2996, 2934, 2835, 1770, 1759, 1512, 1464, 1421, 1240, 1154, 1142, 860, 754.

ESI-MS calcd for $C_{19}H_{20}NO_4$ [M+H]$^+$ 326.1387; found 326.1385.

M.P. 167-169 °C (lit.[1] 167 °C).

TLC (CH_2Cl_2:MeOH 10:1 v/v): R_f = 0.42.

^{1}H NMR data and M.P. agree with those reported in the literature[2].

Pacodine (**3**):

^{1}H NMR (400 MHz, $CDCl_3$) δ = 8.30 (d, *J* = 5.7 Hz, 1H), 7.54 (d, *J* = 0.8 Hz, 1H), 7.42 (dd, *J* = 6.0, 0.8 Hz, 1H), 7.06 (s, 1H), 6.79 (d, *J* = 2.0 Hz, 1H), 6.69 – 6.65 (m, 1H), 6.63 (d, *J* = 8.2 Hz, 1H), 4.43 (s, 2H), 4.00 (s, 3H), 3.76 (s, 3H), 3.71 (s, 3H).

^{13}C NMR (101 MHz, $CDCl_3$) δ = 157.9, 151.1, 149.0, 147.5, 147.1, 140.1, 133.2, 132.0, 123.7, 120.6, 119.1, 111.9, 111.2, 107.9, 105.0, 56.1, 55.9, 55.8, 41.6.

IR (neat, v_{max}/cm^{-1}) 2996, 2936, 2836, 1770, 1760, 1512, 1479, 1433, 1239, 1153, 1050, 1028, 860, 754.

ESI-MS calcd for $C_{19}H_{20}NO_4$ [M+H]$^+$ 326.1387; found 326.1381.

M.P. 163-165 °C (lit.[1] 163-165 °C).

TLC (CH_2Cl_2:MeOH 10:1 v/v): R_f = 0.45.

^{1}H data and M.P. agree with those reported in the literature[2].

Synthesis of (+)-Biotin-2-(methylamino)ethanol amide (**6**):

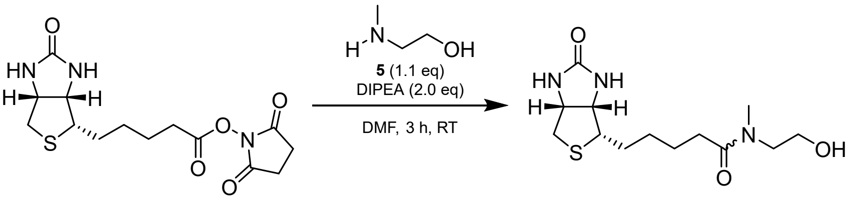

4 **6**

To a solution of (+)-Biotin *N*-hydroxysuccinimide ester (**4**) (500 mg, 1.47 mmol, 1.0 eq) in DMF (41.8 ml, 0.035 M) was added 2-(methylamino)ethanol (**5**) (0.13 ml, 1.61 mmol, 1.1 eq) and DIPEA (0.51 ml, 2.93 mmol, 2.0 eq) at RT. The reaction was stopped after 3 h and concentrated in vacuo at 60 °C. The crude mixture was purified by flash column chromatography (SiO_2; DCM:MeOH 10:1 + 0.1% formic acid) resulting in (+)-Biotin-2-(methylamino)ethanol amide (**6**) as a withe powder (402.9 mg, 0.134 mmol, 91.0% yield, 1.2:1.0 cis/trans amide). Reaction conditions were adapted from literature[3].

(+)-Biotin-2-(methylamino)ethanol amide (**6**): (signals of the minor isomer are indicated with an asterisk)

Note: *Some ^{13}C signals were assigned using 2D HSQC and HMBC NMR spectra.*

^{1}H NMR (500 MHz, DMSO-d$_6$) δ = 6.43 (s, 1H, 1H*), 6.35 (s, 1H, 1H*), 4.79 (t, *J* = 5.4 Hz, 1H), 4.61 (t, *J* = 5.5 Hz, 1H*), 4.33 – 4.27 (m, 1H, 1H*), 4.13 (m, 1H, 1H*), 3.51 (q, *J* = 5.6 Hz, 2H), 3.44 (q, *J* = 5.9 Hz, 2H*), 3.35 – 3.30 (m, 2H, 2H*), 3.10 (m, 1H, 1H*), 2.98 (s, 3H), 2.85 – 2.80 (m, 1H, 1H*), 2.80 (s, 3H*), 2.57 (d, *J* = 12.4 Hz, 1H, 1H*), 2.32 (t, *J* = 7.5 Hz, 2H), 2.26 (t, *J* = 7.4 Hz, 2H*), 1.67 – 1.57 (m, 1H, 1H*), 1.55 – 1.42 (m, 3H, 3H*), 1.39 – 1.27 (m, 2H, 2H*).

^{13}C NMR (126 MHz, DMSO-d$_6$) δ = 172.1 (CON), 171.9 (CON*), 162.7 (C, C*), 61.1 (CH, CH*), 59.2 (CH, CH*), 58.7 (CH_2*), 58.6z (CH_2), 55.5 (CH), 55.5 (CH*), 51.3 (CH_2), 49.7 (CH_2*), 39.5 (CH_2, CH_2*), 36.2 (CH_3), 33.1 (CH_3*), 32.4 (CH_2*), 31.9 (CH_2), 28.4 (CH_2), 28.3 (CH_2*), 28.2 (CH_2), 28.2 (CH_2*), 25.0 (CH_2), 24.6 (CH_2*).

IR (neat, v_{max}/cm^{-1}) 3270, 2939, 2830, 1687, 1660, 1625, 1464, 1355, 1074, 1053, 860, 773.

ESI-MS calcd for $C_{13}H_{23}N_3NaO_3S$ $[M+Na]^+$ 324.1353; found 324.1353.

M.P. decomposition at ≥ 200 °C.

TLC (CH_2Cl_2:MeOH 10:1 v/v + 0.1% formic acid): R_f = 0.19.

$[\alpha]^{26}_D$ = 52.2(c = 0.25, DMSO).

<u>Synthesis of *N*-(2-((1-(3,4-dimethoxybenzyl)-6-methoxyisoquinolin-7-yl)oxy)ethyl)-*N*-methyl-5-((3a*S*,4*S*,6a*R*)-2-oxohexahydro-1*H*-thieno[3,4-d]imidazol-4-yl)pentanamide (**7**):</u>

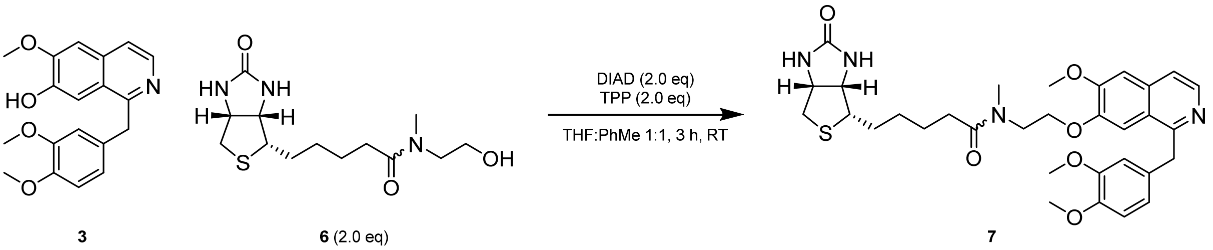

3 **6** (2.0 eq) **7**

To a solution of triphenylphosphine (33.9 mg, 0.13 mmol, 2.0 eq) in THF:PhMe (1:1, 1.6 ml, 0.04 M) was added diisopropyl azodicarboxylate (25.3 ml, 0.13 mmol, 2.0 eq) and Pacodine (**3**) (21.0 mg, 0.065 mmol, 1.0 eq) at RT resulting in a yellow solution. After 10 min, alcohol **6** (38.9 mg, 0.13 mmol, 2.0 eq) was added. After 3 h, the reaction was quenched by addition of 0.1 M aq. HCl solution. The acidic mixture was washed three times with EtOAc, basified by addition of sat. aq. $NaHCO_3$ solution and extracted three times with EtOAc. The combined organic layers were dried over $MgSO_4$, filtered and concentrated. The crude mixture was purified by preparative TLC (SiO_2; DCM:MeOH 10:1) resulting in **7** as a beige wax (14.2 mg, 0.022 mmol, 33.6% yield, 1.0:3.0 *cis*/*trans* amide) along with reisolated Pacodine (**3**) (4.6 mg, 0.014 mmol, 21.9% yield). Reaction conditions were adapted from literature[4].

N-(2-((1-(3,4-dimethoxybenzyl)-6-methoxyisoquinolin-7-yl)oxy)ethyl)-*N*-methyl-5-((3a*S*,4*S*,6a*R*)-2-oxohexahydro-1H-thieno[3,4-d]imidazol-4-yl)pentanamide (**7**): (signals of the minor isomer are indicated with an asterisk)

Note: *Some ^{13}C signals were assigned using 2D HSQC and HMBC NMR spectra.*

^{1}H NMR (500 MHz, $CDCl_3$) δ = 8.36 (d, *J* = 5.8 Hz, 1H*), 8.34 (d, *J* = 5.9 Hz, 1H), 7.56 – 7.45 (m, 2H, 1H*), 7.34 (s, 1H*), 7.07 (s, 1H), 7.07 (s, 1H*), 6.98 (s, 1H), 6.91 – 6.84 (m, 1H, 1H*), 6.81 – 6.72 (m, 1H, 2H*), 5.55 (s, 1H*), 5.52 (s, 1H), 5.03 (s, 1H*), 4.99 (s, 1H), 4.68 – 4.55 (m, 2H, 2H*), 4.50 – 4.40 (m, 1H, 1H*), 4.32 – 4.20 (m, 3H, 1H*), 4.09 (t, *J* = 5.3 Hz, 2H*), 3.99 (s, 3H), 3.96 (s, 3H*), 3.85 – 3.74 (m, 8H, 8H*), 3.17 (s, 3H), 3.12 – 3.04 (m, 1H, 1H*), 3.00 (s, 3H*), 2.92 – 2.85 (m, 1H*), 2.84 (dd, *J* = 12.9, 5.0 Hz, 1H), 2.70 (d, *J* = 13.0 Hz, 1H*), 2.67 (d, *J* = 12.8 Hz, 1H), 2.50 (t, *J* = 7.4 Hz, 2H*), 2.36 (t, *J* = 7.3 Hz, 2H), 1.79 – 1.58 (m, 4H, 4H*), 1.50 – 1.40 (m, 2H, 2H*).

^{13}C NMR (126 MHz, $CDCl_3$) δ = 173.6 (CO*), 173.4 (CO), 163.3 (CO*), 163.2 (CO), 157.6 (C, C*), 152.8 (C, C*),149.3 (C*), 149.2 (C), 147.8 (C, C*), 141,1 (CH, CH*), 133.7(C), 133.9(C*), 132.0 (C, C*), 122.9 (C), 122.8 (C*), 120.9 (CH), 120.6 (CH*), 119.40 (CH, CH*), 112.3 (CH), 112.2 (CH*), 111.4 (CH), 111.4 (CH*), 105.9 (CH*), 105.8 (CH*), 105.7 (2CH), 67.6 (CH_2), 66.3 (CH_2*), 62.0 (CH*), 61.9 (CH), 60.2 (CH*), 60.2 (CH), 56.3 (CH_3), 56.2 (CH_3*), 56.1 (CH_3), 56.1 (CH_3*), 56.1 (CH_3*), 56.0 (CH_3), 55.4 (CH*), 55.3 (CH), 49.0 (CH_2*), 47.7 (CH_2), 41,9 (CH_2, CH_2*) 40.7 (CH_2*), 40.6 (CH_2), 37.8 (CH_3), 34.0 (CH_3*), 33.0 (CH_2), 32.5 (CH_2*), 28.6 (CH_2*), 28.5 (CH_2*), 28.4 (CH_2), 28.3 (CH_2), 25.2 (CH_2*), 24.7 (CH_2).

IR (neat, ν_{max}/cm^{-1}) 3250, 2930, 2855, 1702, 1628, 1510, 1478, 1464, 1434, 1269, 1234, 1160, 1140, 1027, 753.

ESI-MS calcd for $C_{32}H_{41}N_4O_6S$ $[M+H]^+$ 609.2741; found 609.2737.

TLC (CH_2Cl_2:MeOH 10:1 v/v): R_f = 0.18.

$[\alpha]^{24}_{D}$ = 22.1(c = 1.0, $CHCl_3$).

Following the NMR spectra highlighting key ^{1}H NOESY cross-peaks identifying the predominant amide geometry in solution and confirming the attachment site of the biotinylated side chain to Papaverine.

NMR spectral data

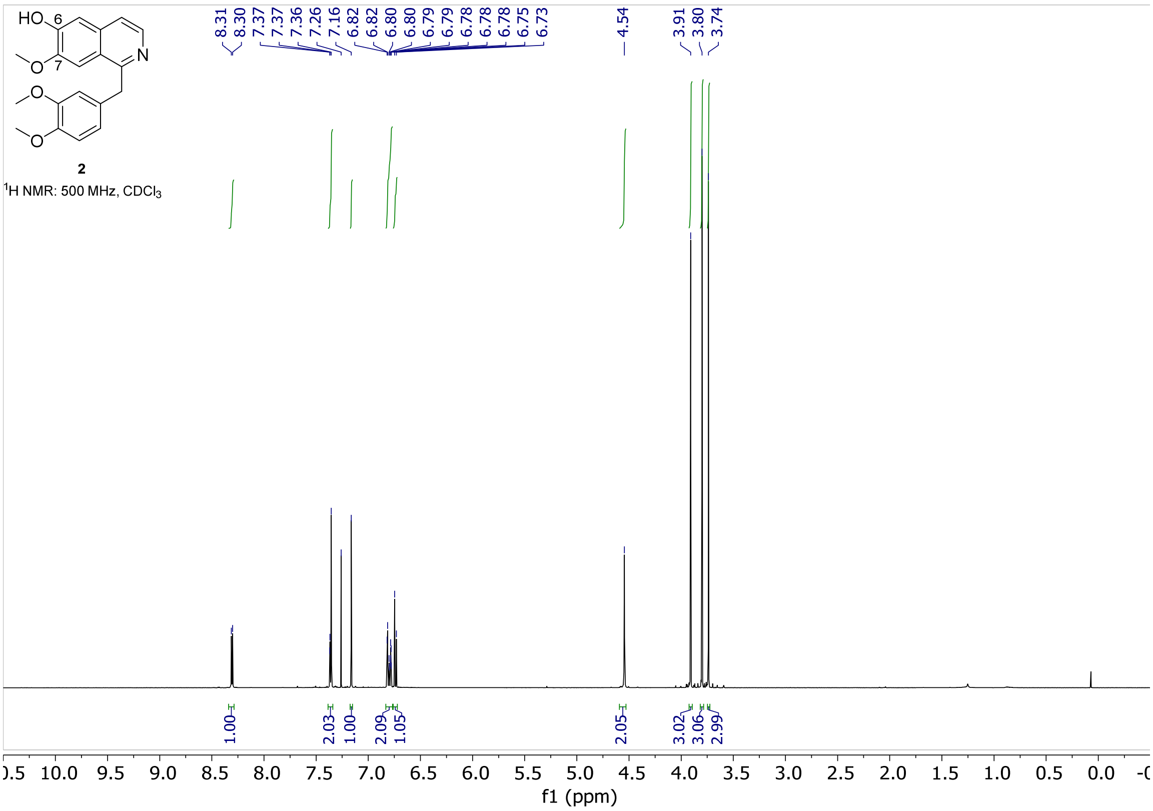

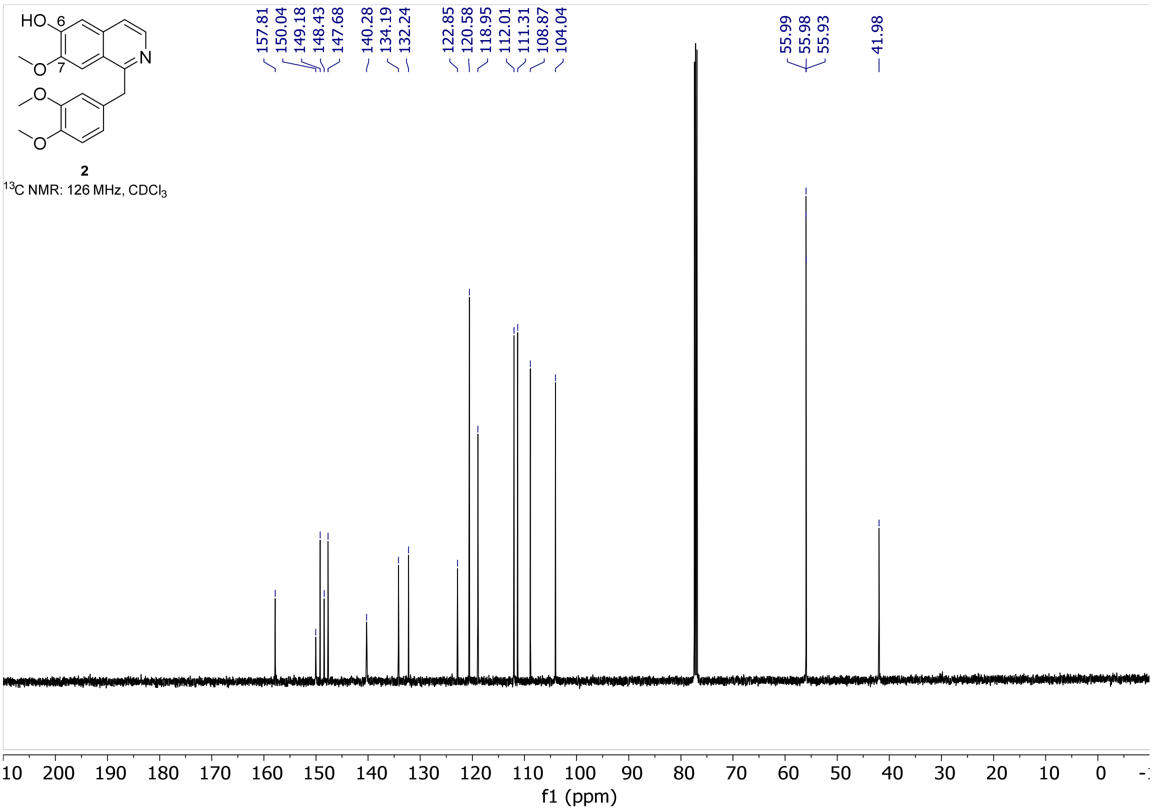

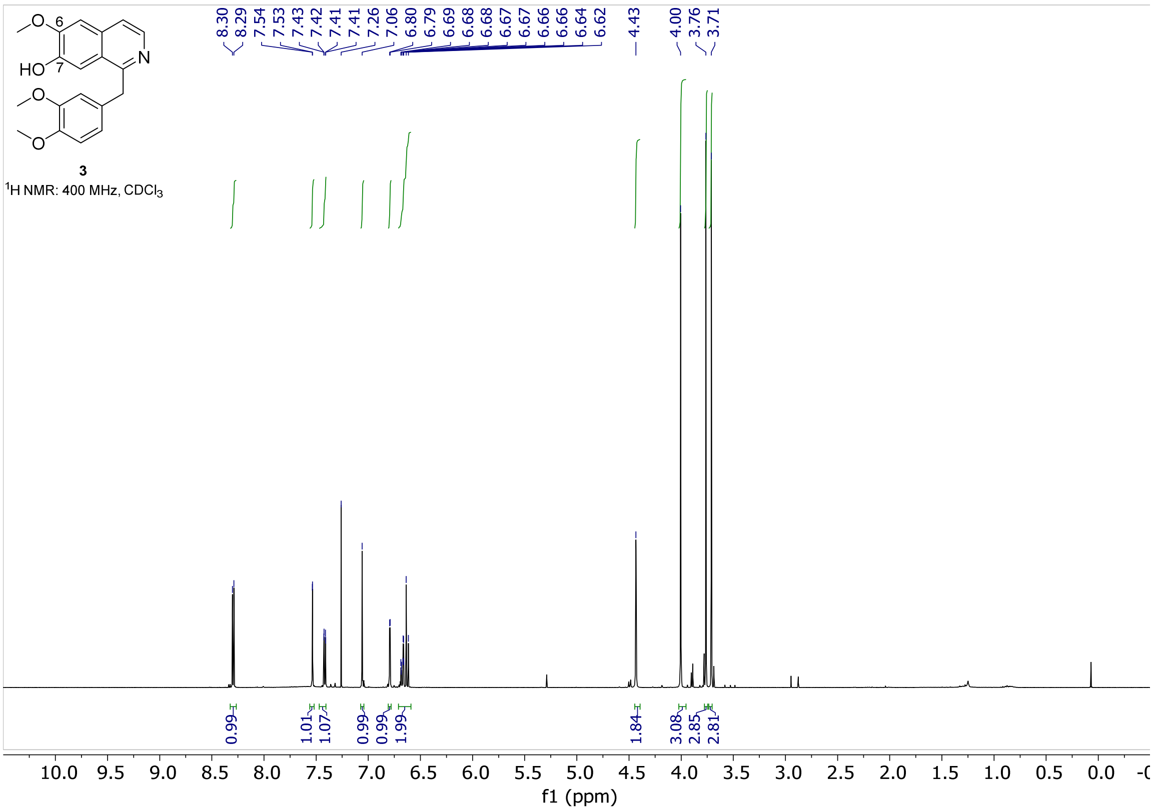

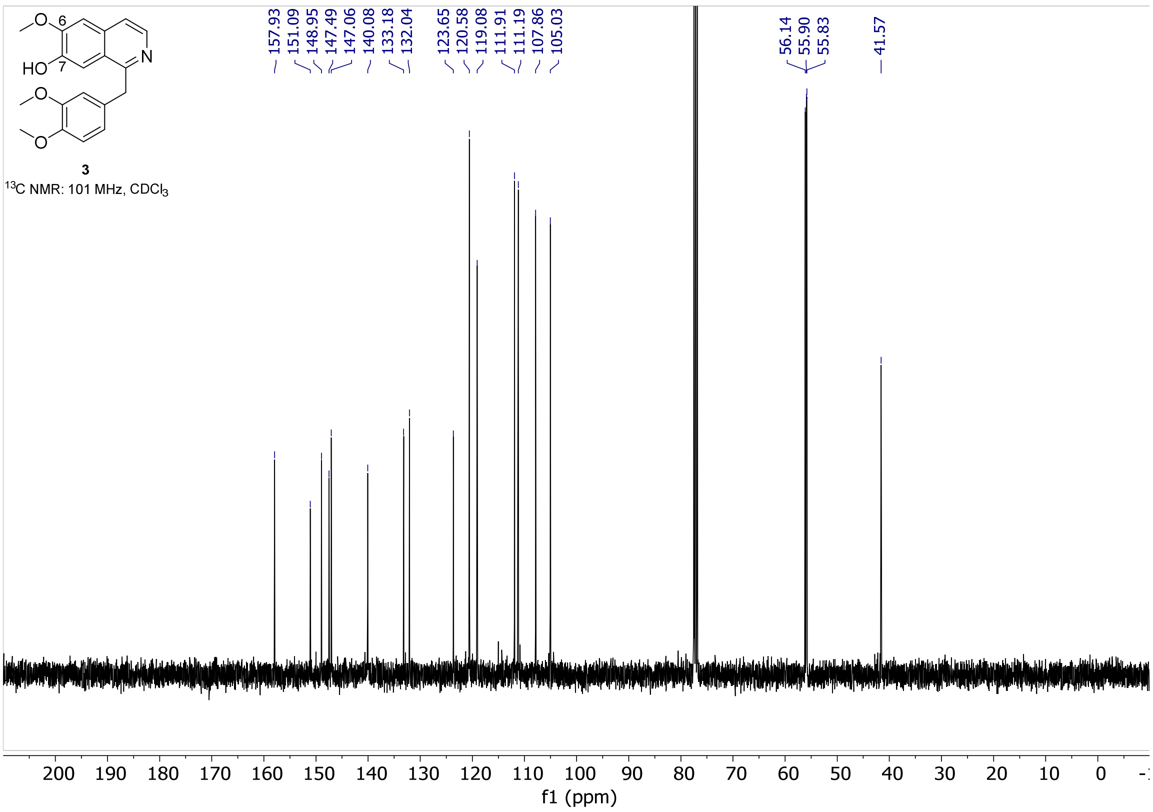

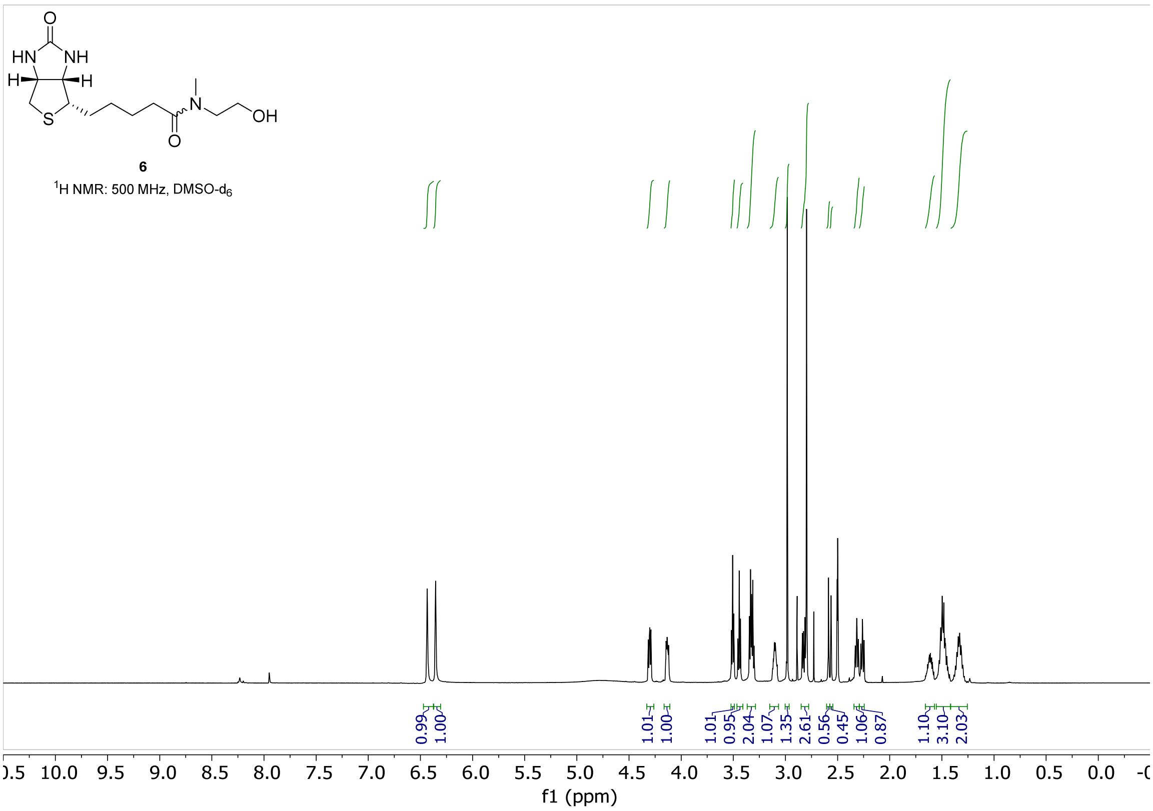
6
^{1}H NMR: 500 MHz, DMSO-d$_6$
0.99
1.00
1.01
1.00
1.01
0.95
2.04
1.07
1.35
2.61
0.56
0.45
1.06
0.87
1.10
3.10
2.03
f1 (ppm)

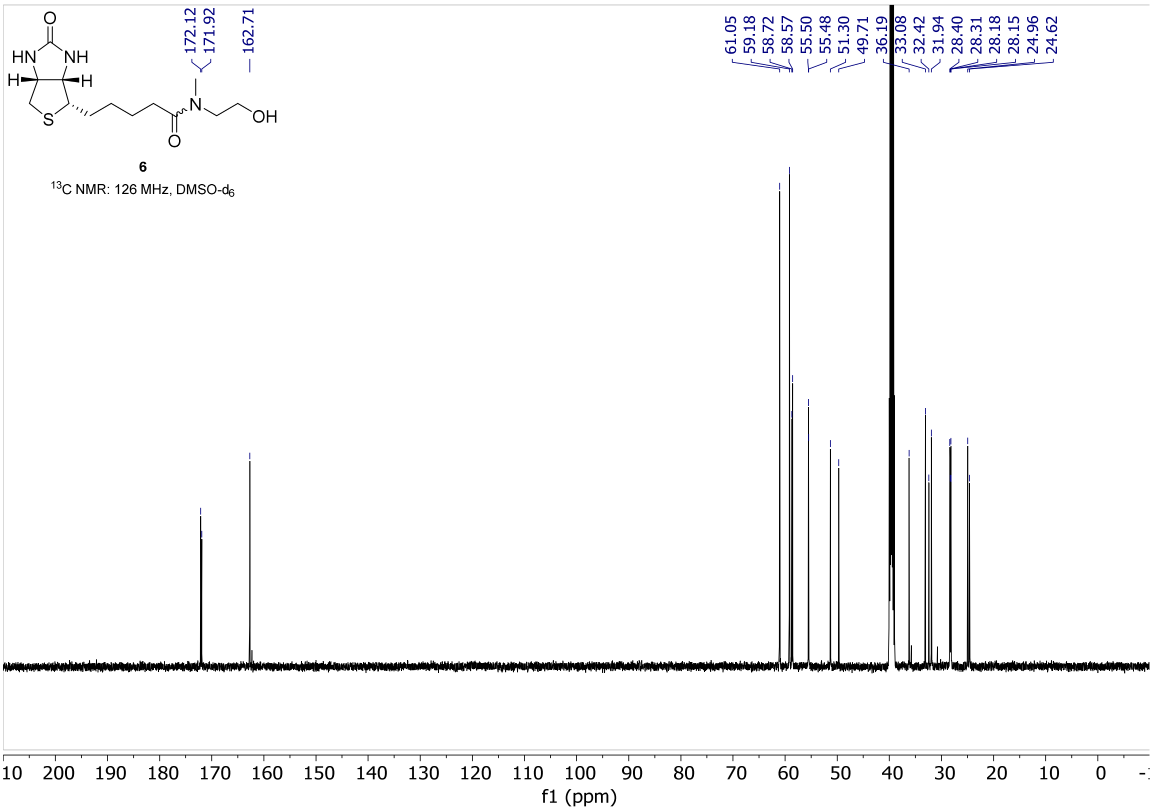
6
^{13}C NMR: 126 MHz, DMSO-d$_6$
172.12
171.92
162.71
61.05
59.18
58.72
58.57
55.50
55.48
51.30
49.71
36.19
33.08
32.42
31.94
28.40
28.31
28.18
28.15
24.96
24.62
f1 (ppm)

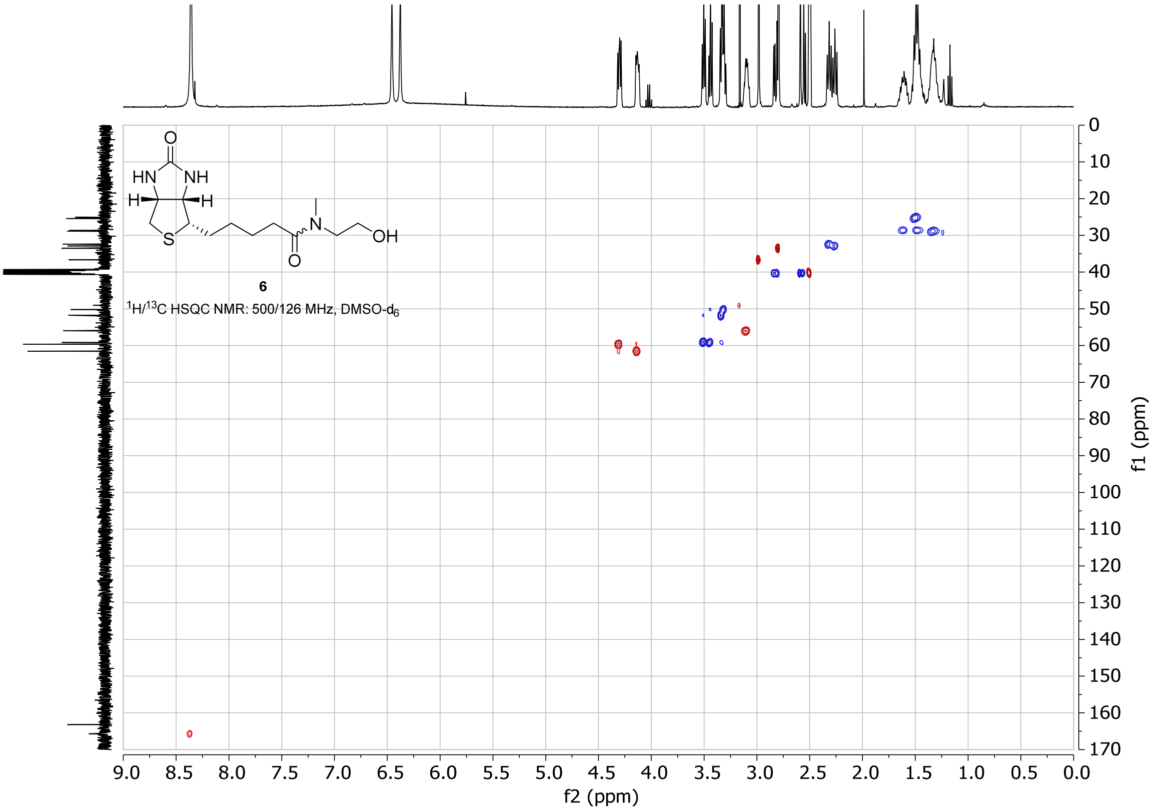
6
$^1H/^{13}C$ HSQC NMR: 500/126 MHz, DMSO-d_6
f2 (ppm)
f1 (ppm)

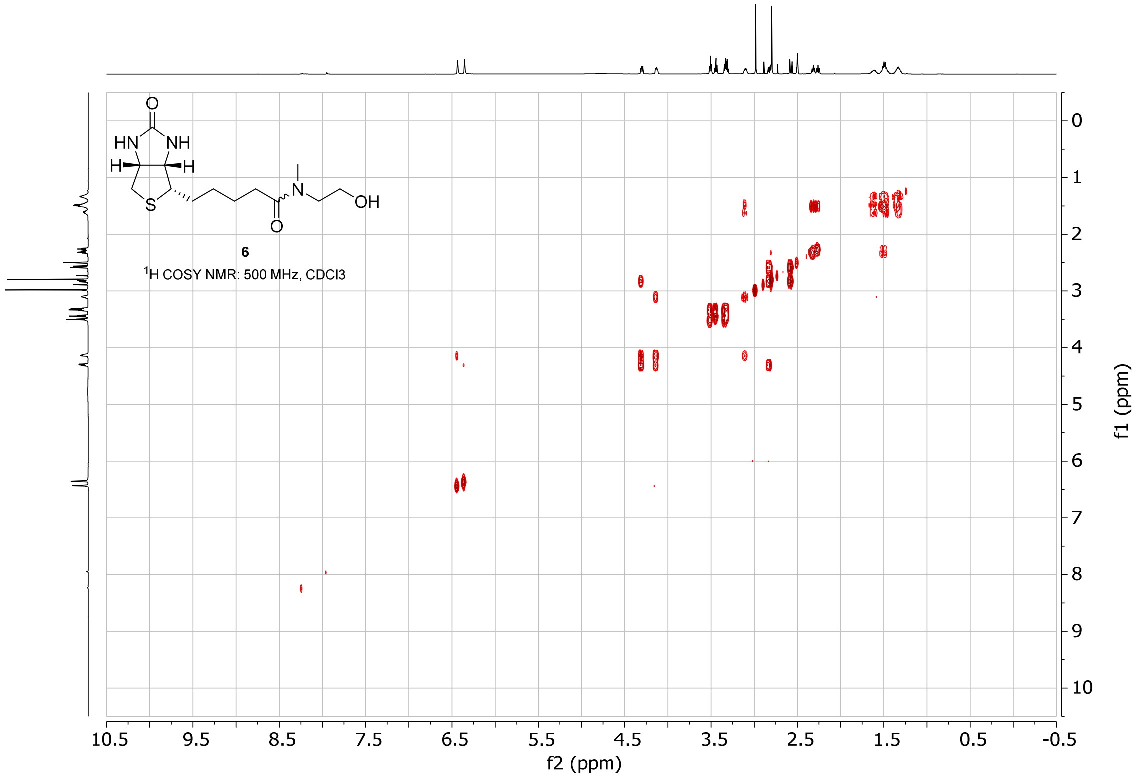
6
^1H COSY NMR: 500 MHz, CDCl3
f2 (ppm)
f1 (ppm)

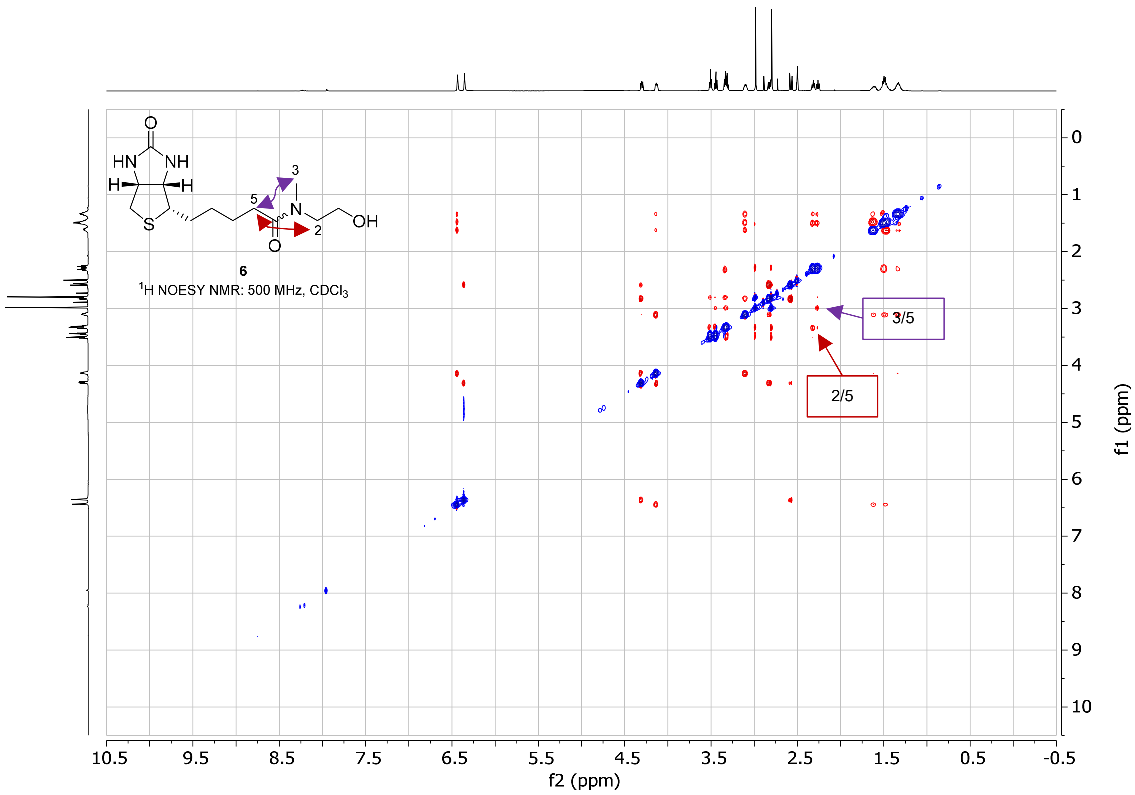
O
HN
NH
H
H
S
5
3
N
2
OH
O
6
1H NOESY NMR: 500 MHz, CDCl3
3/5
2/5
f1 (ppm)
f2 (ppm)

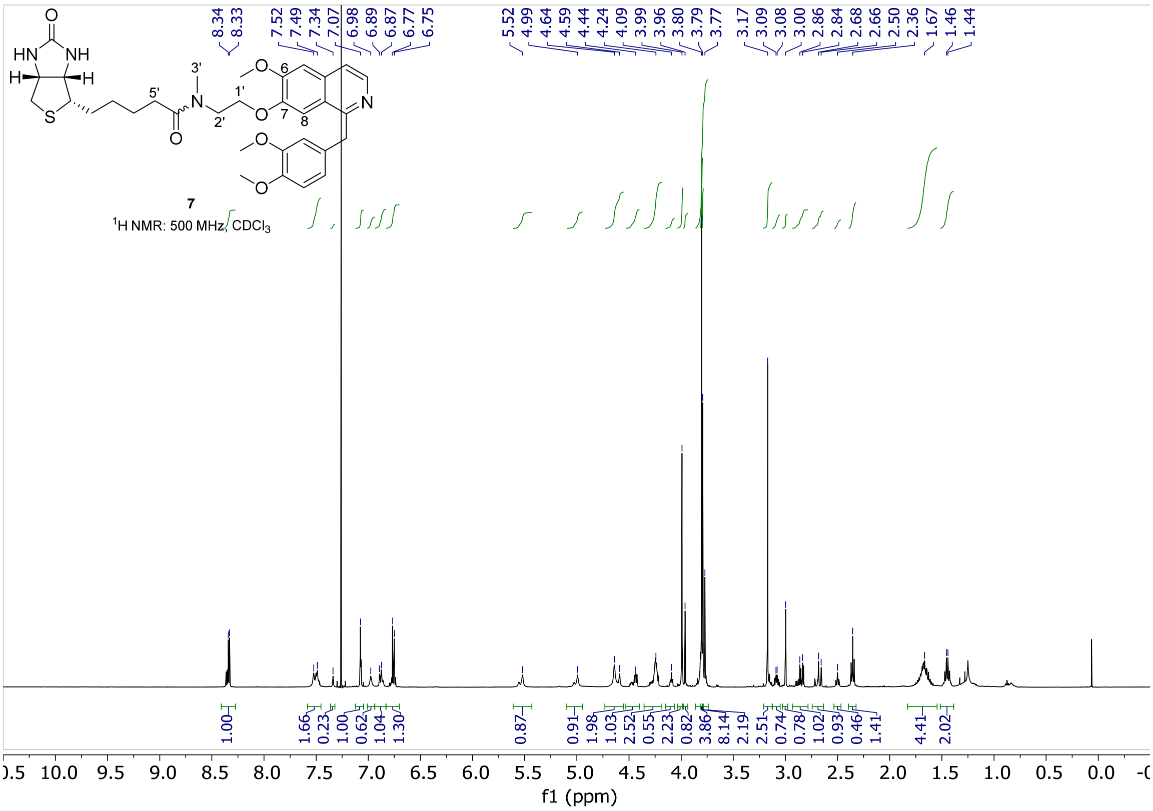

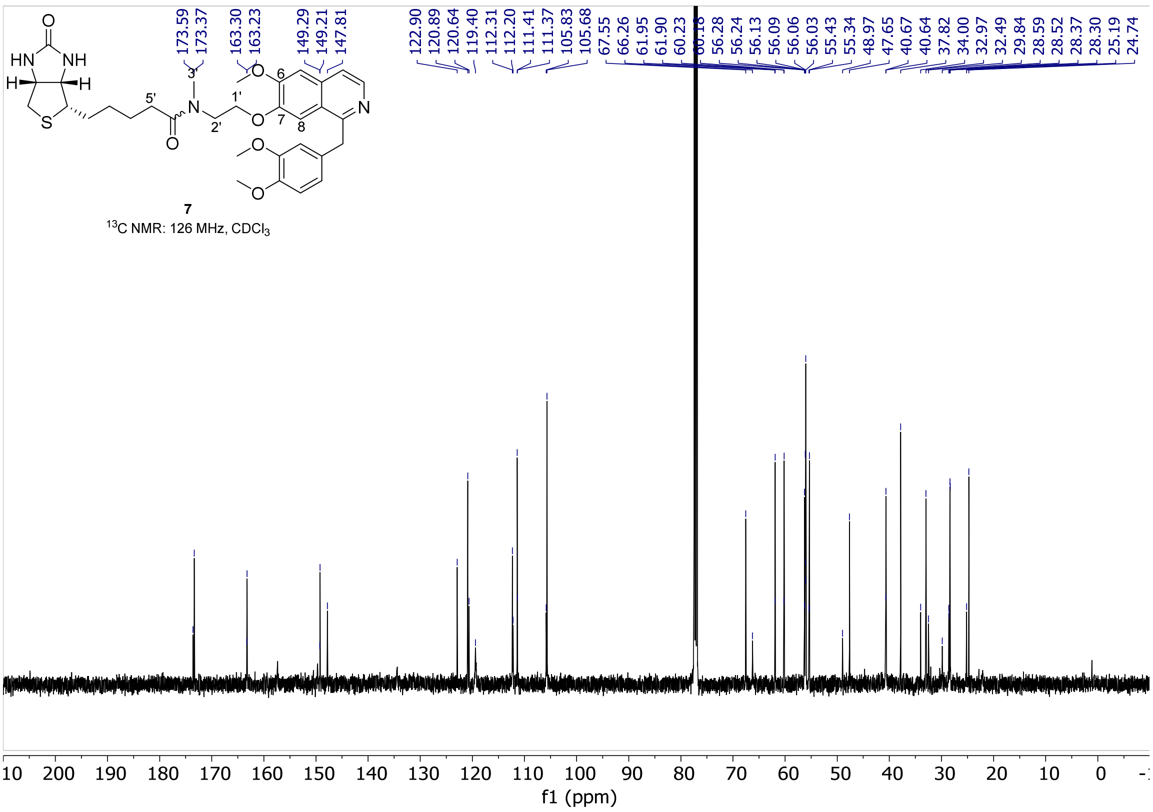

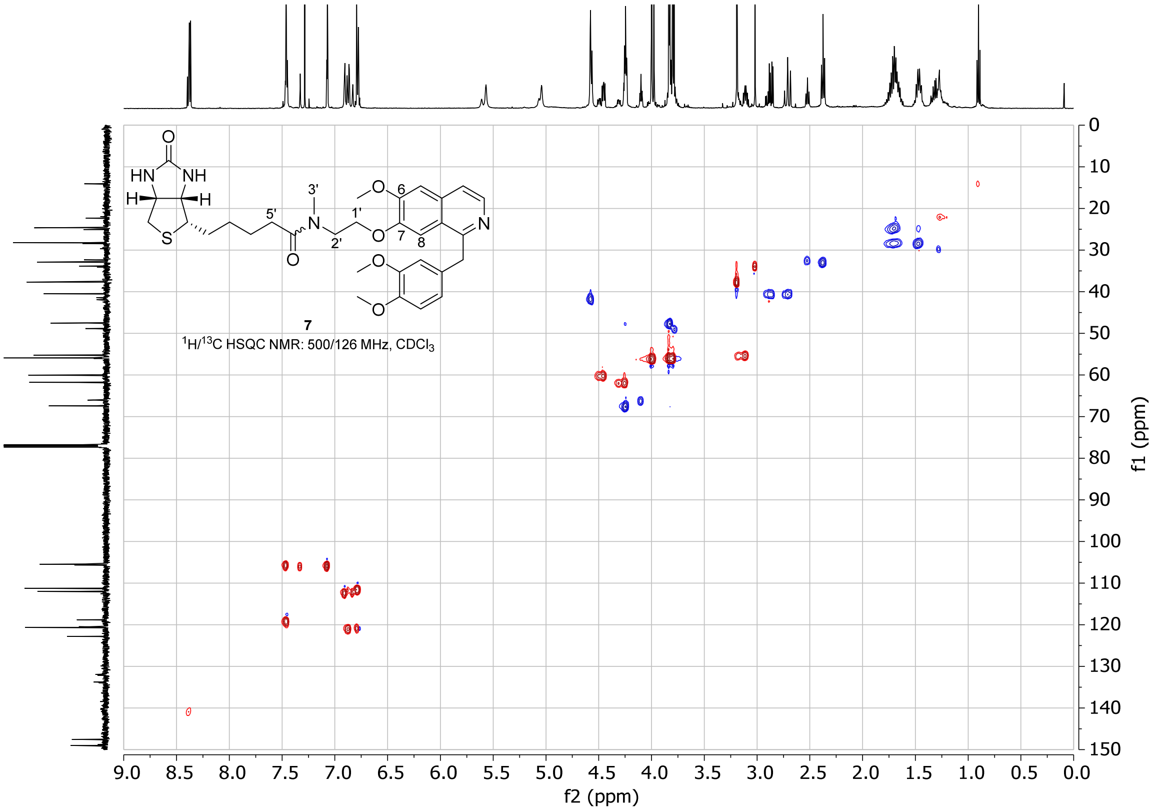
7
$^1H/^{13}C$ HSQC NMR: 500/126 MHz, $CDCl_3$
f1 (ppm)
f2 (ppm)

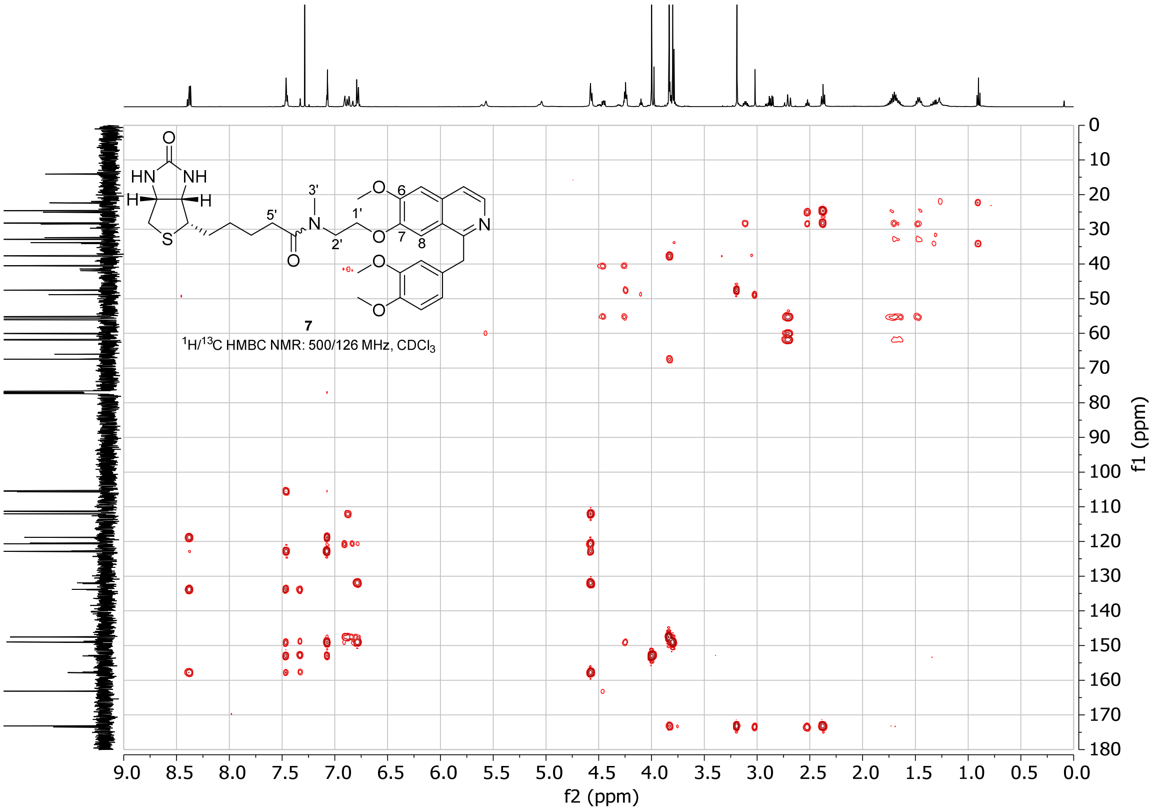
7
$^1H/^{13}C$ HMBC NMR: 500/126 MHz, $CDCl_3$
f1 (ppm)
f2 (ppm)

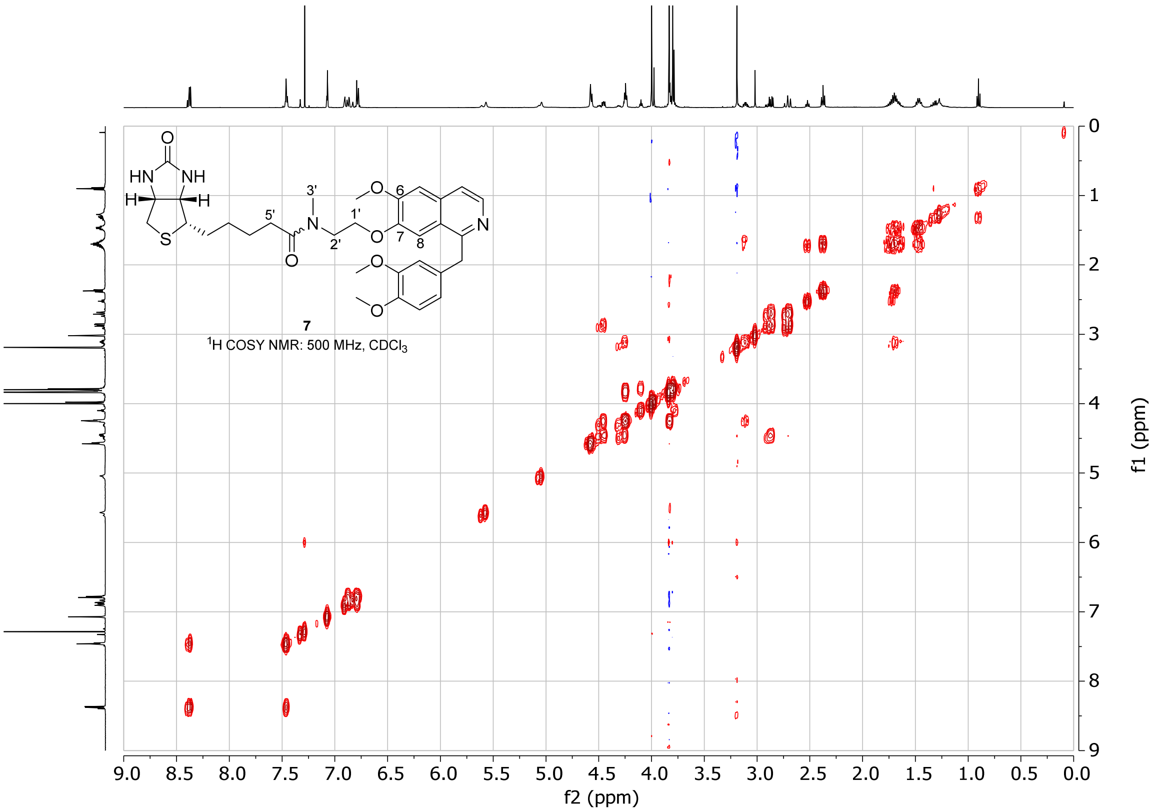
7
^{1}H COSY NMR: 500 MHz, CDCl$_3$
f2 (ppm)
f1 (ppm)

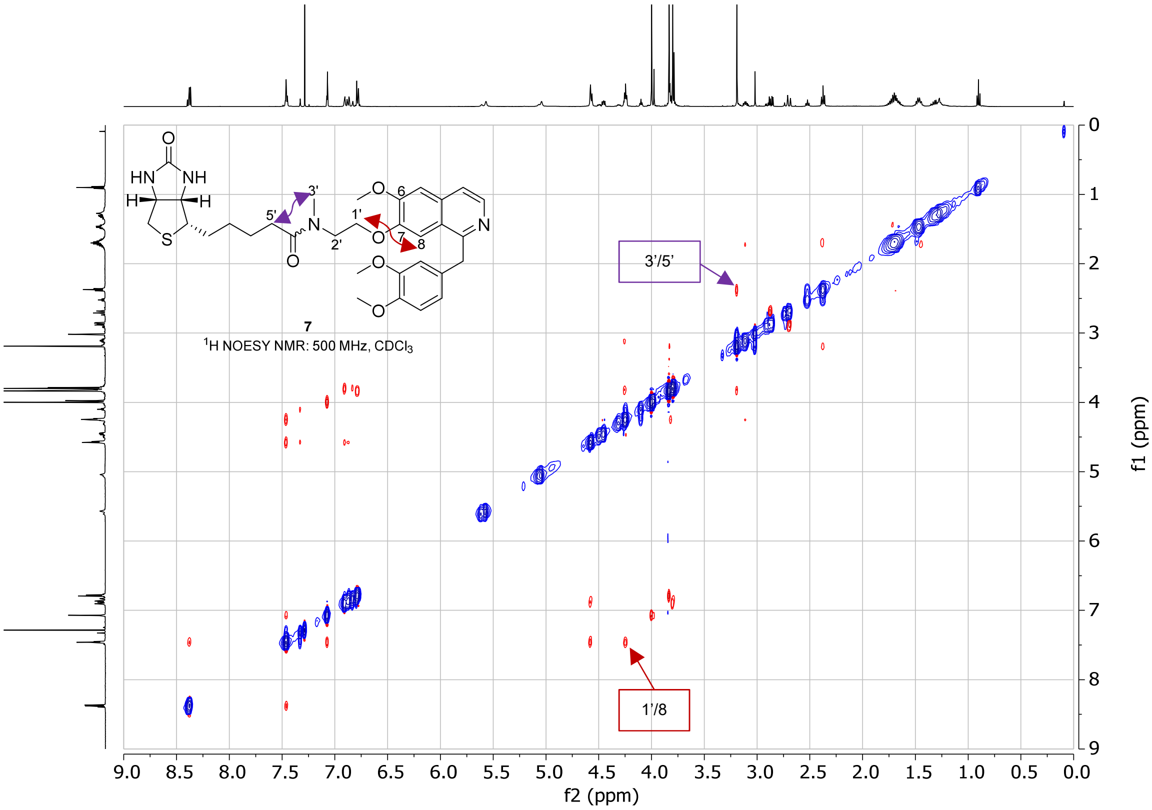
7
^{1}H NOESY NMR: 500 MHz, CDCl$_3$
3'/5'
1'/8
f2 (ppm)
f1 (ppm)

SUPPLEMENTARY REFERENCES

1. Feutrill GI, Mirrington RN. Demethylation of aryl methyl ethers with thioethoxide ion in dimethyl formamide. *Tetrahedron Lett*, 1327-1328 (1970).

2. Brochmann-Hanssen E, Hirai K. Opium alkaloids. VII. Isolation of a new benzylisoquinoline alkaloid. Synthesis and NMR studies of papaveroline trimethyl ethers. *J Pharm Sci* **57**, 940-943 (1968).

3. Hussey SL, Muddana SS, Peterson BR. Synthesis of a beta-estradiol-biotin chimera that potently heterodimerizes estrogen receptor and streptavidin proteins in a yeast three-hybrid system. *J Am Chem Soc* **125**, 3692-3693 (2003).

4. Adams DJ*, et al.* Synthesis, cellular evaluation, and mechanism of action of piperlongumine analogs. *Proc Natl Acad Sci U S A* **109**, 15115-15120 (2012).
