# Supplementary material for: Grainyhead 1 acts as a drug-inducible conserved transcriptional regulator linked to insulin signaling and lifespan
Source: Nat Commun. 2022 Jan 10;13:107. doi: 10.1038/s41467-021-27732-4 (PMC8748497; doi:10.1038/s41467-021-27732-4)
Supplement: Supplementary file 3 — Description of Additional Supplementary Files [file 41467_2021_27732_MOESM3_ESM.pdf]

SUPPLEMENTARY FILES

Grainyhead 1 acts as a drug-inducible conserved transcriptional regulator linked to insulin signaling and lifespan

Giovanna Grigolon, Elisa Araldi, Reto Erni, Jia Yee Wu, Carolin Thomas, Marco La Fortezza, Beate Laube, Doris Pöhlmann, Markus Stoffel, Kim Zarse, Erick M. Carreira, Michael Ristow, Fabian Fischer

Corresponding author: Michael Ristow (email: michael-ristow@ethz.ch)

File Name: Supplementary Data 1
File Type: Excel Workbook (*.xlsx)
Description: *C. elegans* lifespan assay statistics. Detailed data and statistical analyses of all lifespan assays and repeats thereof performed throughout this study.

File Name: Supplementary Data 2
File Type: Excel Workbook (*.xlsx)
Description: *C. elegans* RNA-Seq data. Genes differentially overexpressed in *grh-1* OEx vs. wild-type nematodes with *P*-value < 0.05, as identified by RNA-Seq.

File Name: Supplementary Data 3
File Type: Excel Workbook (*.xlsx)
Description: GRH-1 DNA-binding sites analysis. GRH-1 binding sites in the predicted promoters of the differentially overexpressed genes listed in Supplementary Data 2, as determined using the FIMO algorithm from the MEME Suite and the BiocManager::TFBSTools function in R. Genes in the MAP kinase pathway are highlighted in red font.

File Name: Supplementary Data 4
File Type Excel Workbook (*.xlsx)
Description: Primary compound screening results. Results of the primary screening of all 2,560 compounds from "The Spectrum Collection" on the GRHL and ARE/NRF2 luciferase reporter cell lines.

File Name: Supplementary Data 5
File Type: Excel Workbook (*.xlsx)
Description: Human GRHL1-interacting proteins. All proteins selectively co-purifying with 3xFLAG-6xHis tagged wild-type human GRHL1, as detected by mass spectrometry.

File Name: Supplementary Data 6
File Type: Portable Document Format (*.pdf)
Description: Annotated MS/MS spectra of GRHL1 post-translational modifications associated with papaverine treatment. MS/MS spectra of all GRHL1$^{WT\text{-}TAG}$ peptides corresponding to post-translational modifications exclusively identified in papaverine-treated samples according to PEAKS X search engine results.

File Name: Supplementary Data 7
File Type: Portable Document Format (*.pdf)
Description: XICs of GRHL1 post-translational modifications associated with papaverine treatment. Extracted ion chromatograms (XICs) of the modified peptides identified only in the papaverine-treated samples and not in any of the DMSO control samples according to PEAKS X search engine results. The corresponding unmodified peptides are included, if identified. Raw files were imported into Skyline 21.1.0.146 (https://doi.org/10.1074/mcp.m112.017707), and the precursor MS1 traces were extracted ([M], [M+1], [M+2]). A peptide MS/MS library was build using the identification results from PEAKS and used as a reference for the selection of the correct MS1 traces.
