# Supplementary material for: Grainyhead 1 acts as a drug-inducible conserved transcriptional regulator linked to insulin signaling and lifespan
Source: Nat Commun. 2022 Jan 10;13:107. doi: 10.1038/s41467-021-27732-4 (PMC8748497; doi:10.1038/s41467-021-27732-4)

Supplementary Data 6. Annotated MS/MS spectra of GRHL1 post-translational modifications associated with papaverine treatment.

GRHL1 arginine 9 - dimethylation
MTQEY(+79.97)DNK(+114.04)R(+28.03)PVLVLQNEALYPQR.R

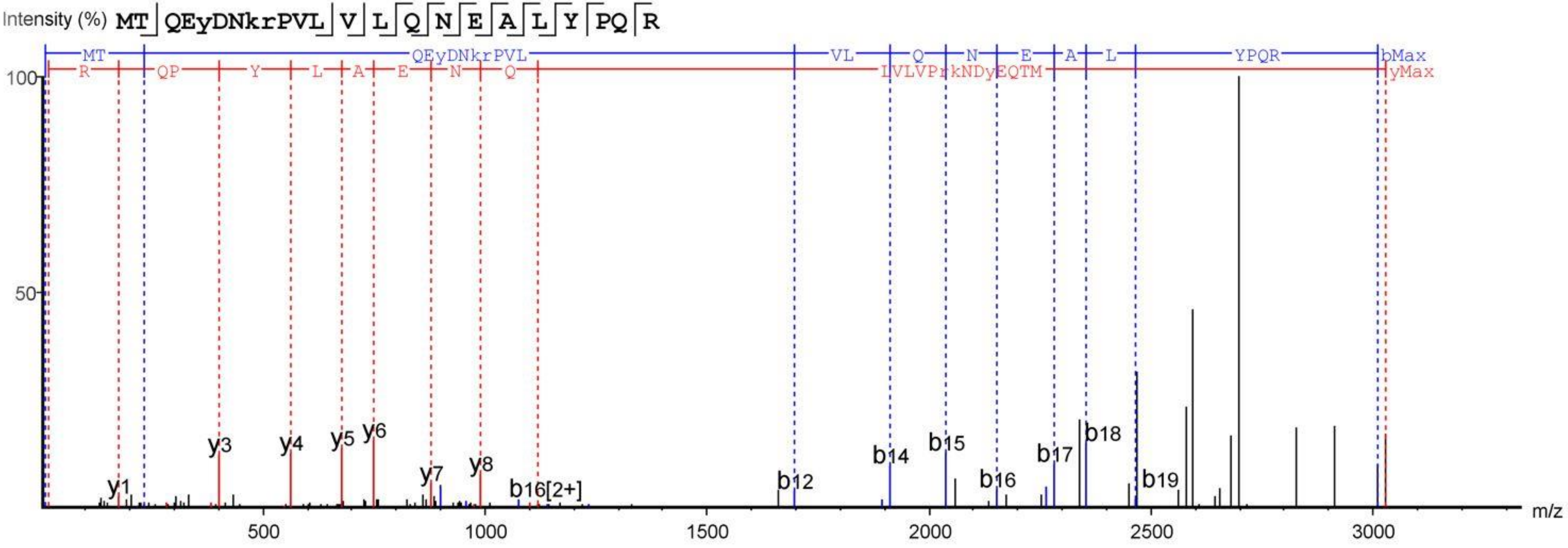

Supplementary Data 6. Annotated MS/MS spectra of GRHL1 post-translational modifications associated with papaverine treatment.

GRHL1 serine 95 - phoshporylation
K.RNS(+79.97)IPIVTEQPLISAGENR.V

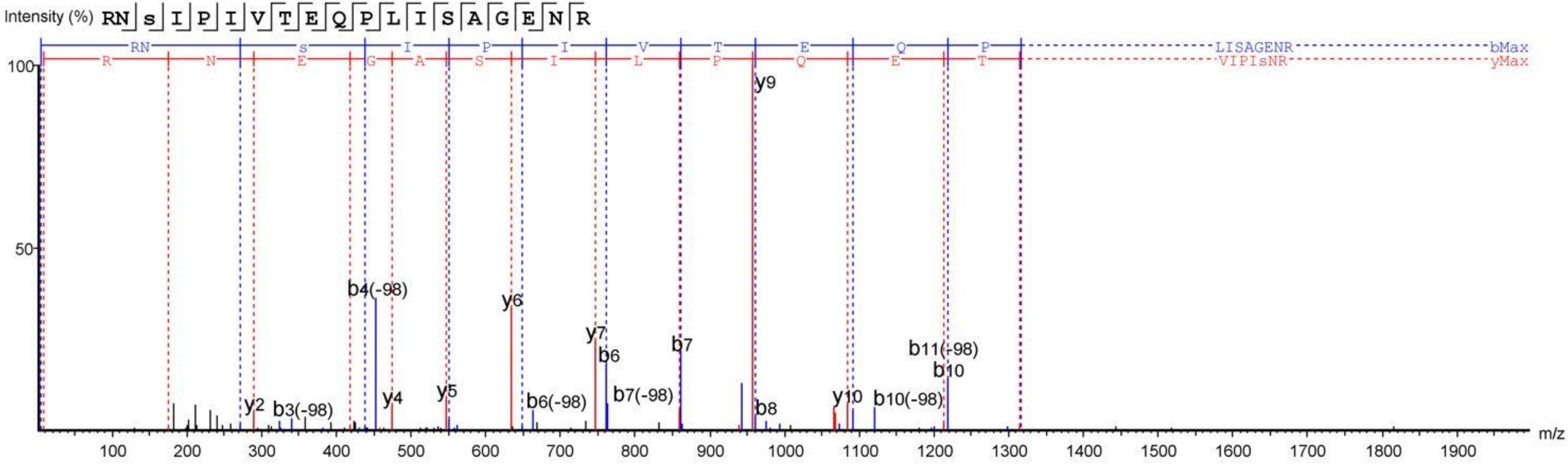

Supplementary Data 6. Annotated MS/MS spectra of GRHL1 post-translational modifications associated with papaverine treatment.

GRHL1 lysine 116 - dimethylation
L.K(+28.03)NVPFNIVLPHGNQLGIDK.R

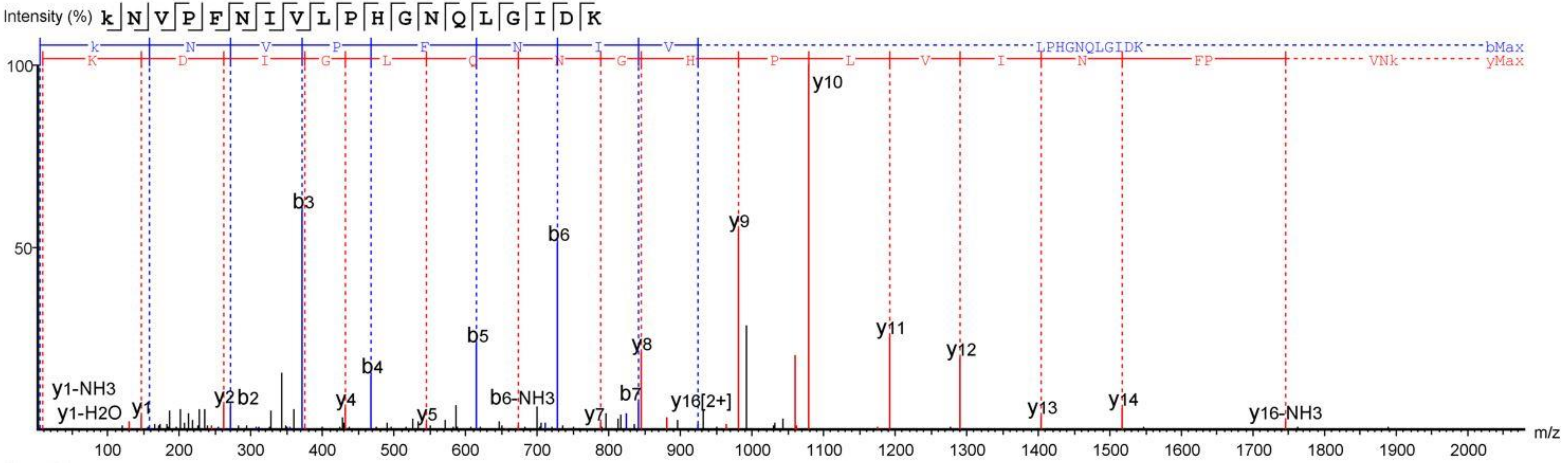

Supplementary Data 6. Annotated MS/MS spectra of GRHL1 post-translational modifications associated with papaverine treatment.

GRHL1 threonine 139 - phosphorylation
D.K(+42.01)RGHLT(+79.97)APDTTVTVSIATMPTHSIK.T

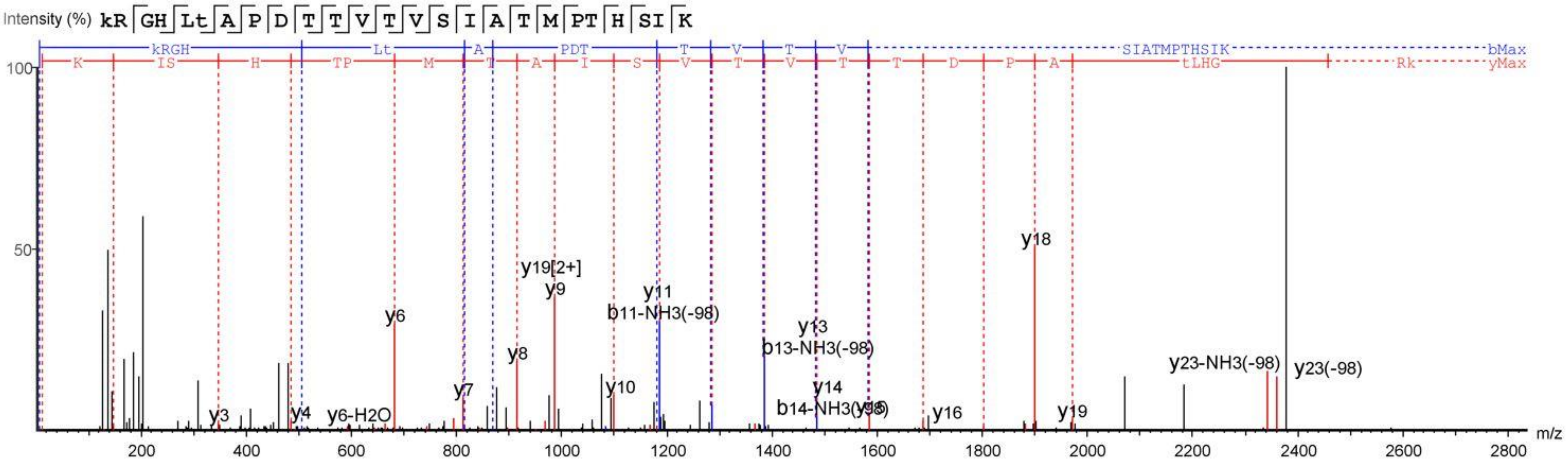

Supplementary Data 6. Annotated MS/MS spectra of GRHL1 post-translational modifications associated with papaverine treatment.

GRHL1 lysine 370 - acetylation
F.TWDINDEAK(+42.01)VFISVNCLSTDFSSQK.G

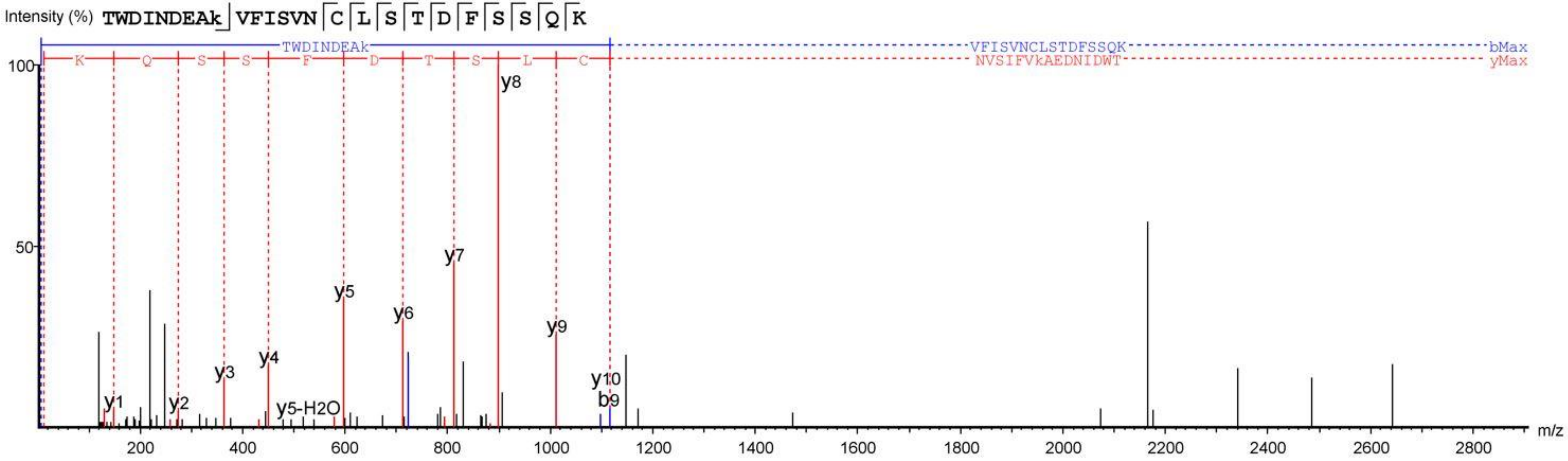

Supplementary Data 6. Annotated MS/MS spectra of GRHL1 post-translational modifications associated with papaverine treatment.

GRHL1 serine 374 - phosphorylation
K.VFIS(+79.97)VNCLSTDFSSQK.G

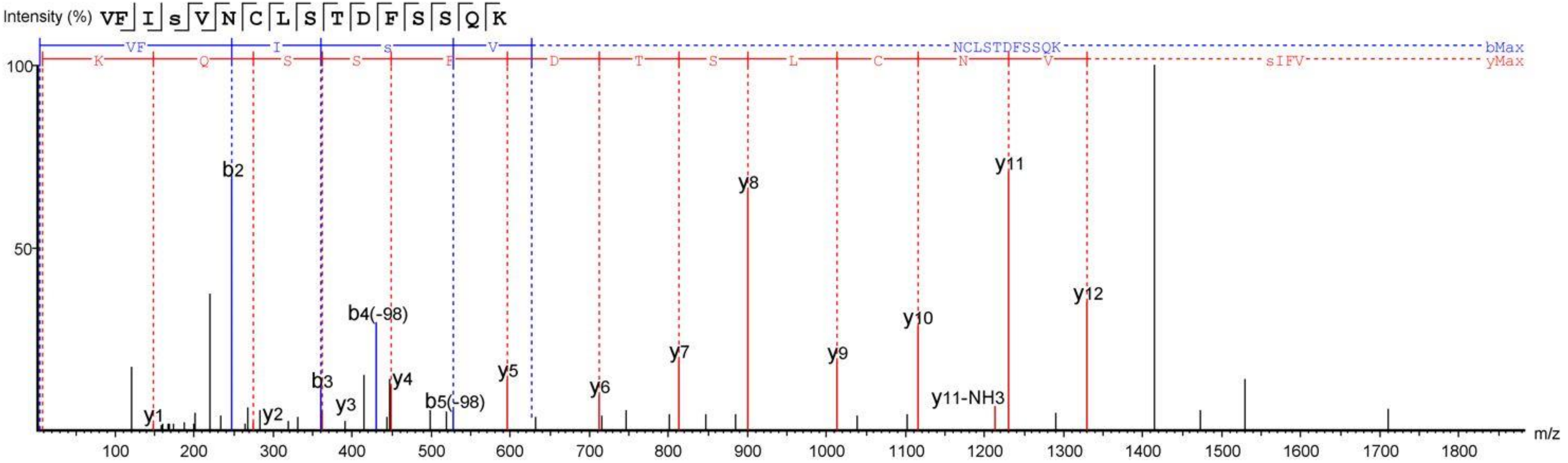

Supplementary Data 6. Annotated MS/MS spectra of GRHL1 post-translational modifications associated with papaverine treatment.

GRHL1 lysine 592 - ubiquitylation
V.NMDDNIVK(+383.23)HYSNEDTFQLQIEEAGGSYK.L

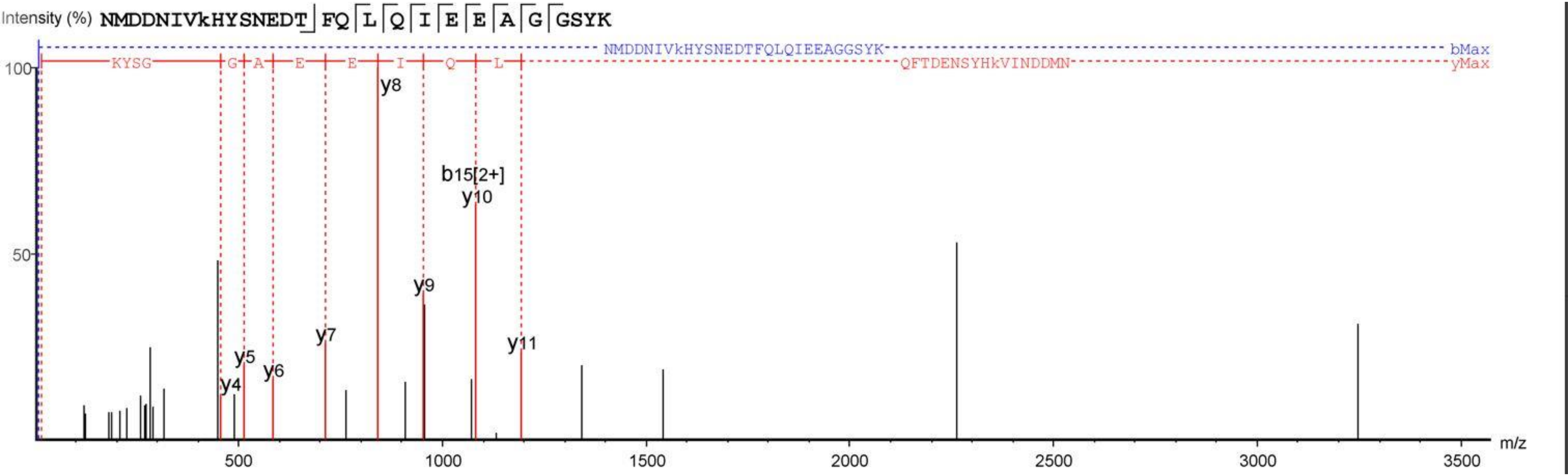

Supplement: Supplementary file 9 — Suppl. Data 6 [file 41467_2021_27732_MOESM9_ESM.pdf]
