# Supplementary material for: Grainyhead 1 acts as a drug-inducible conserved transcriptional regulator linked to insulin signaling and lifespan
Source: Nat Commun. 2022 Jan 10;13:107. doi: 10.1038/s41467-021-27732-4 (PMC8748497; doi:10.1038/s41467-021-27732-4)

Supplementary Data 7. XICs of GRHL1 post-translational modifications associated with papaverine treatment.

R9 - MTQEY**[+80]**DNK**[+114]**R**[+28]**PVLVLQNEALYPQR
D1-3 = DMSO
PH1-3 = Papaverine

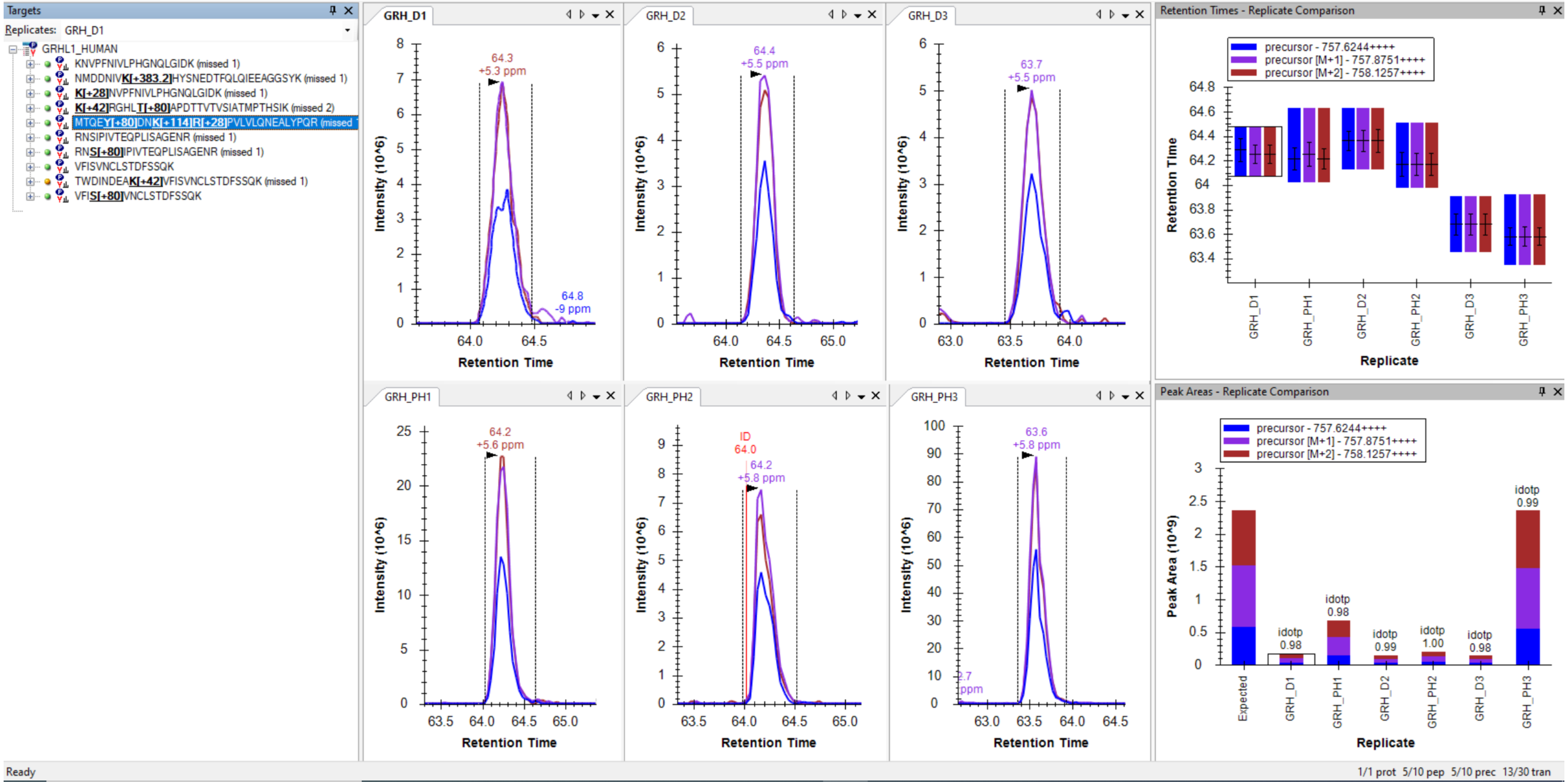

Supplementary Data 7. XICs of GRHL1 post-translational modifications associated with papaverine treatment.

S95 (unmodified) – RNSIPIVTEQPLISAGENR
D1-3 = DMSO
PH1-3 = Papaverine

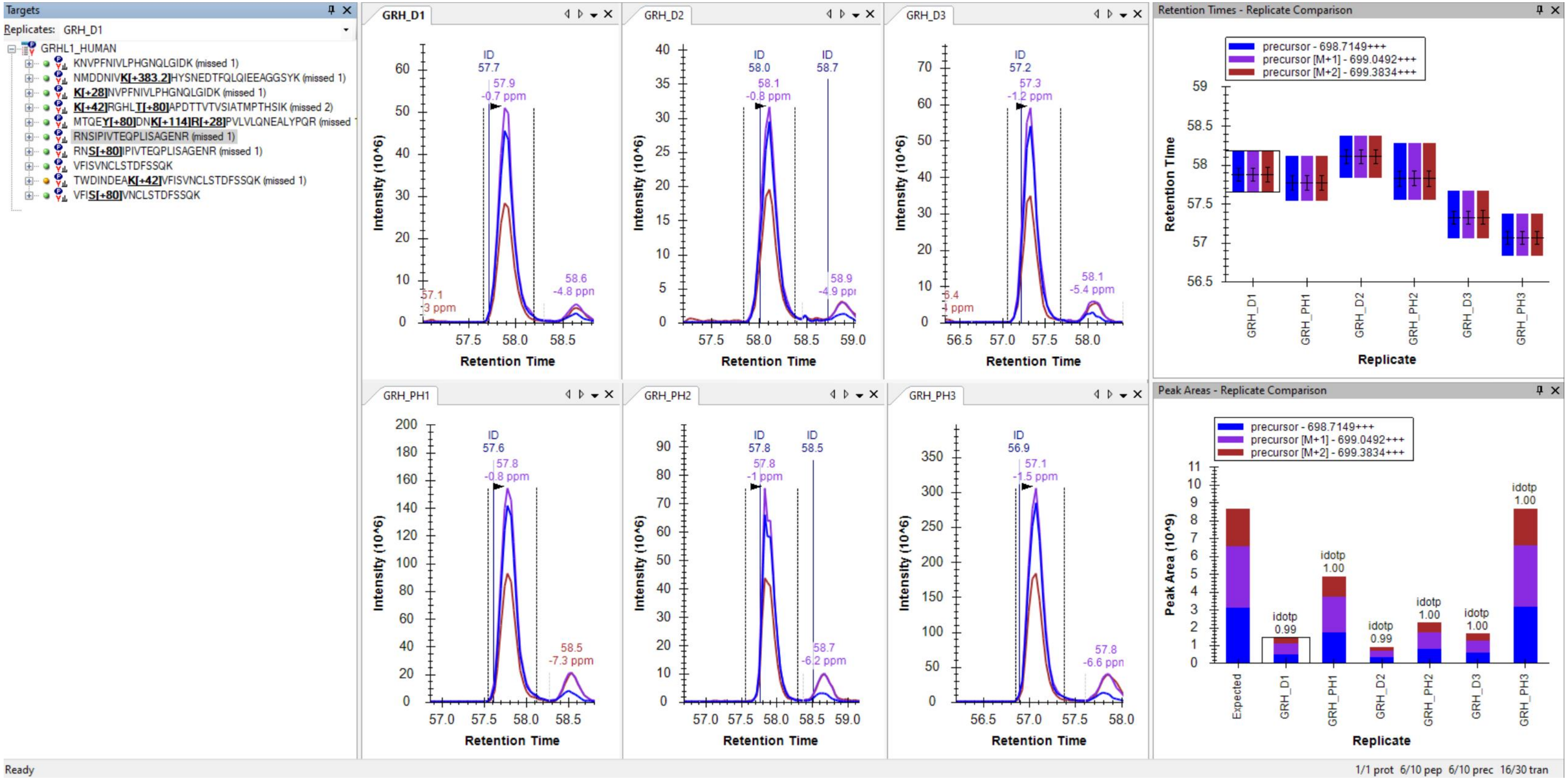

Supplementary Data 7. XICs of GRHL1 post-translational modifications associated with papaverine treatment.

S95 - RNS**[+80]**IPIVTEQPLISAGENR
D1-3 = DMSO
PH1-3 = Papaverine

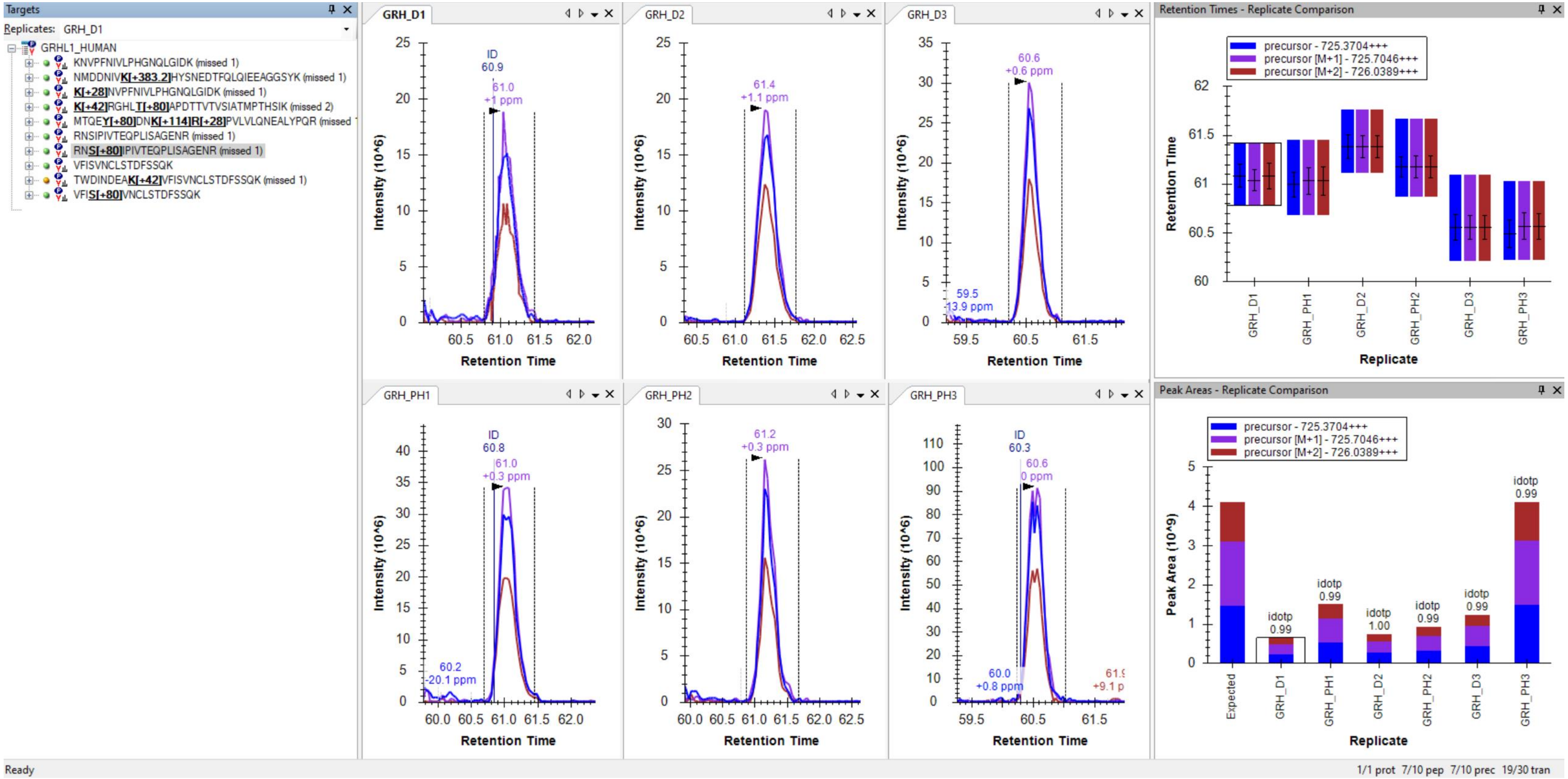

Supplementary Data 7. XICs of GRHL1 post-translational modifications associated with papaverine treatment.

K116 (unmodified) – KNVPFNIVLPHGNQLGIDK
D1-3 = DMSO
PH1-3 = Papaverine

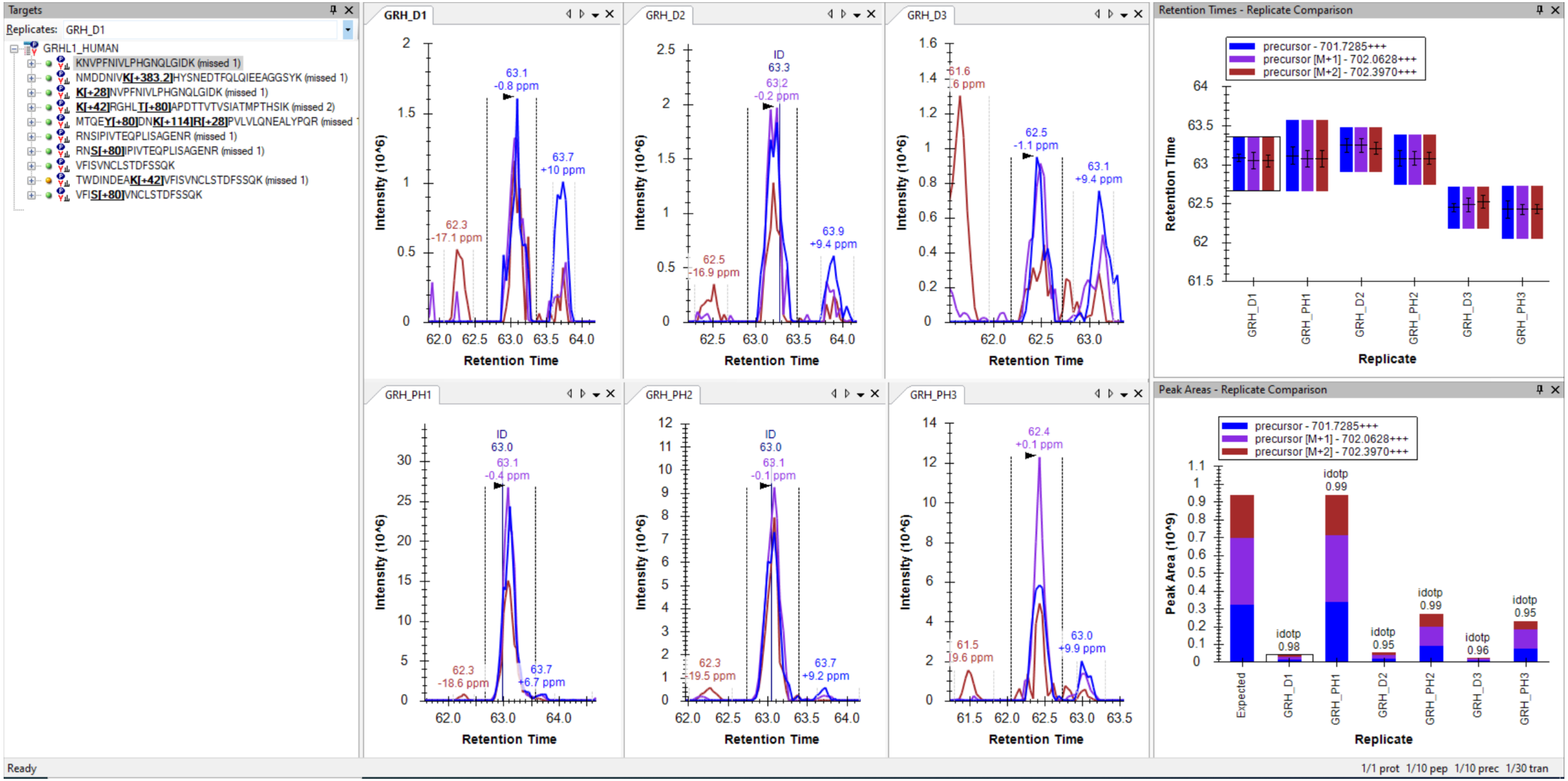

Supplementary Data 7. XICs of GRHL1 post-translational modifications associated with papaverine treatment.

K116 - K[+28]NVPFNIVLPHGNQLGIDK
D1-3 = DMSO
PH1-3 = Papaverine

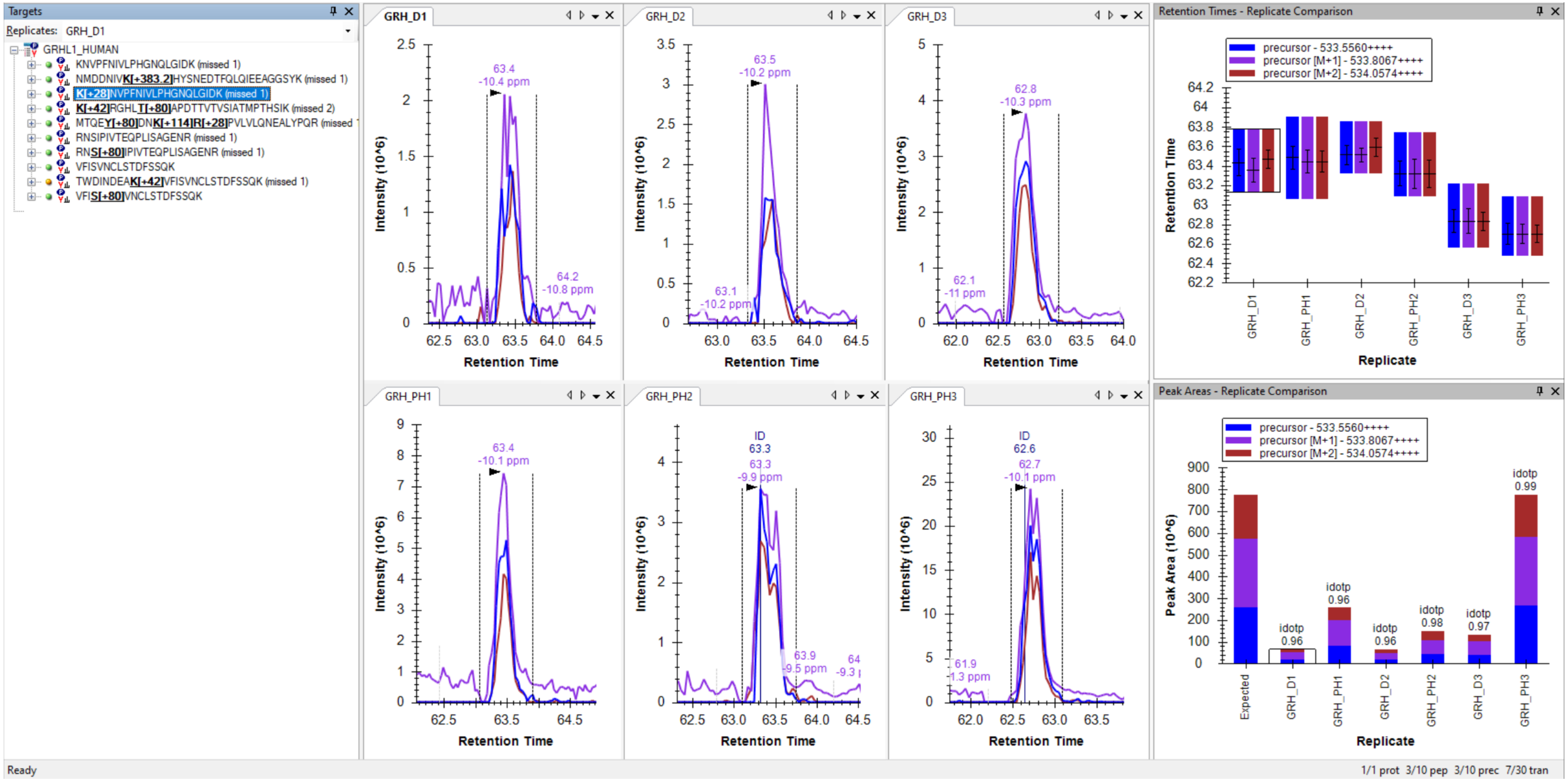

Supplementary Data 7. XICs of GRHL1 post-translational modifications associated with papaverine treatment.

T139 - K**[+42]**RGHLT**[+80]**APDTTVTVSIATMPTHSIK
D1-3 = DMSO
PH1-3 = Papaverine

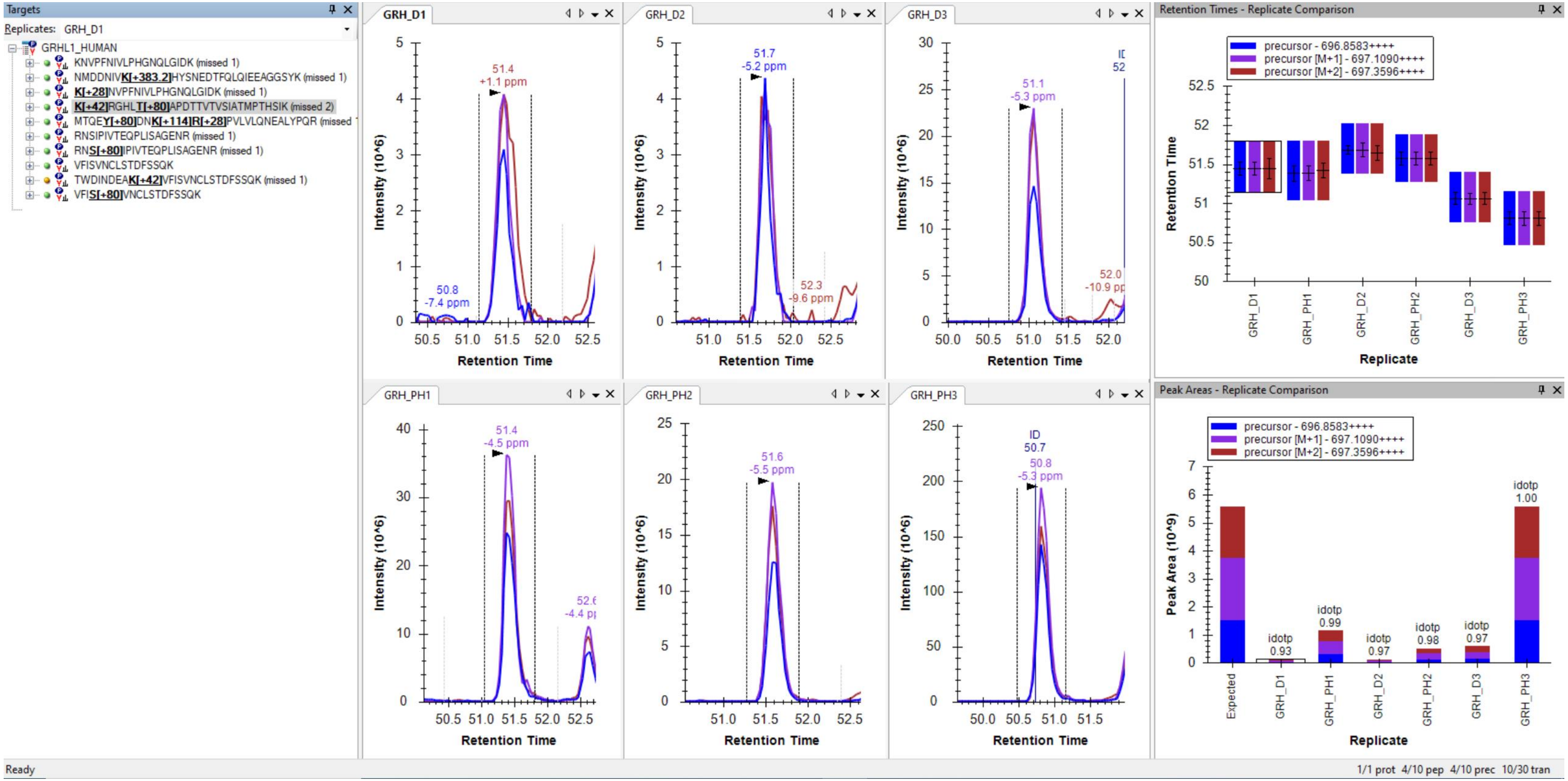

Supplementary Data 7. XICs of GRHL1 post-translational modifications associated with papaverine treatment.

K370 – TWDINDEAK**[+42]**VFISVNCLSTDFSSQK
D1-3 = DMSO
PH1-3 = Papaverine

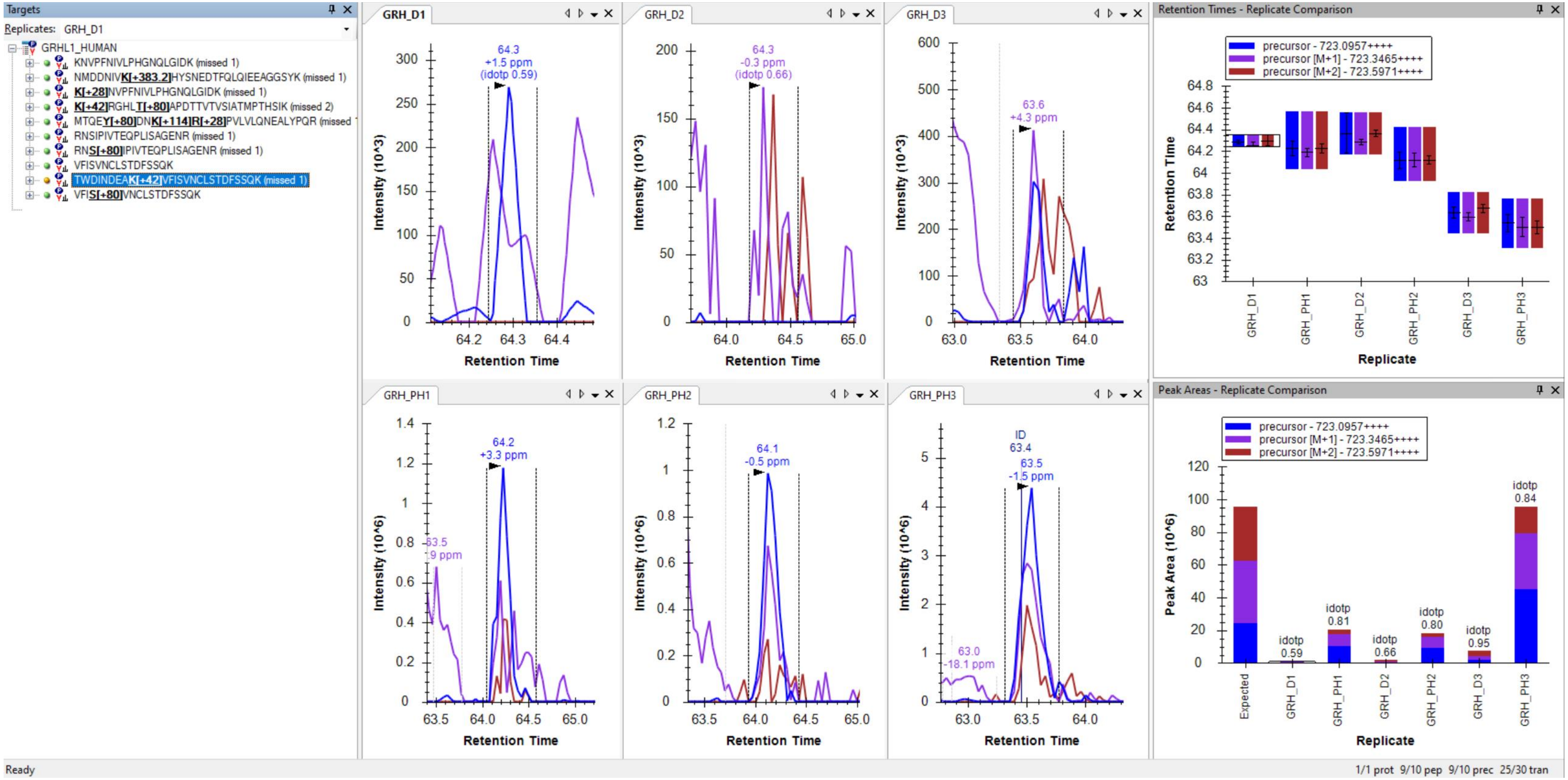

Supplementary Data 7. XICs of GRHL1 post-translational modifications associated with papaverine treatment.

S374 (unmodified) – VFISVNCLSTDFSSQK
D1-3 = DMSO
PH1-3 = Papaverine

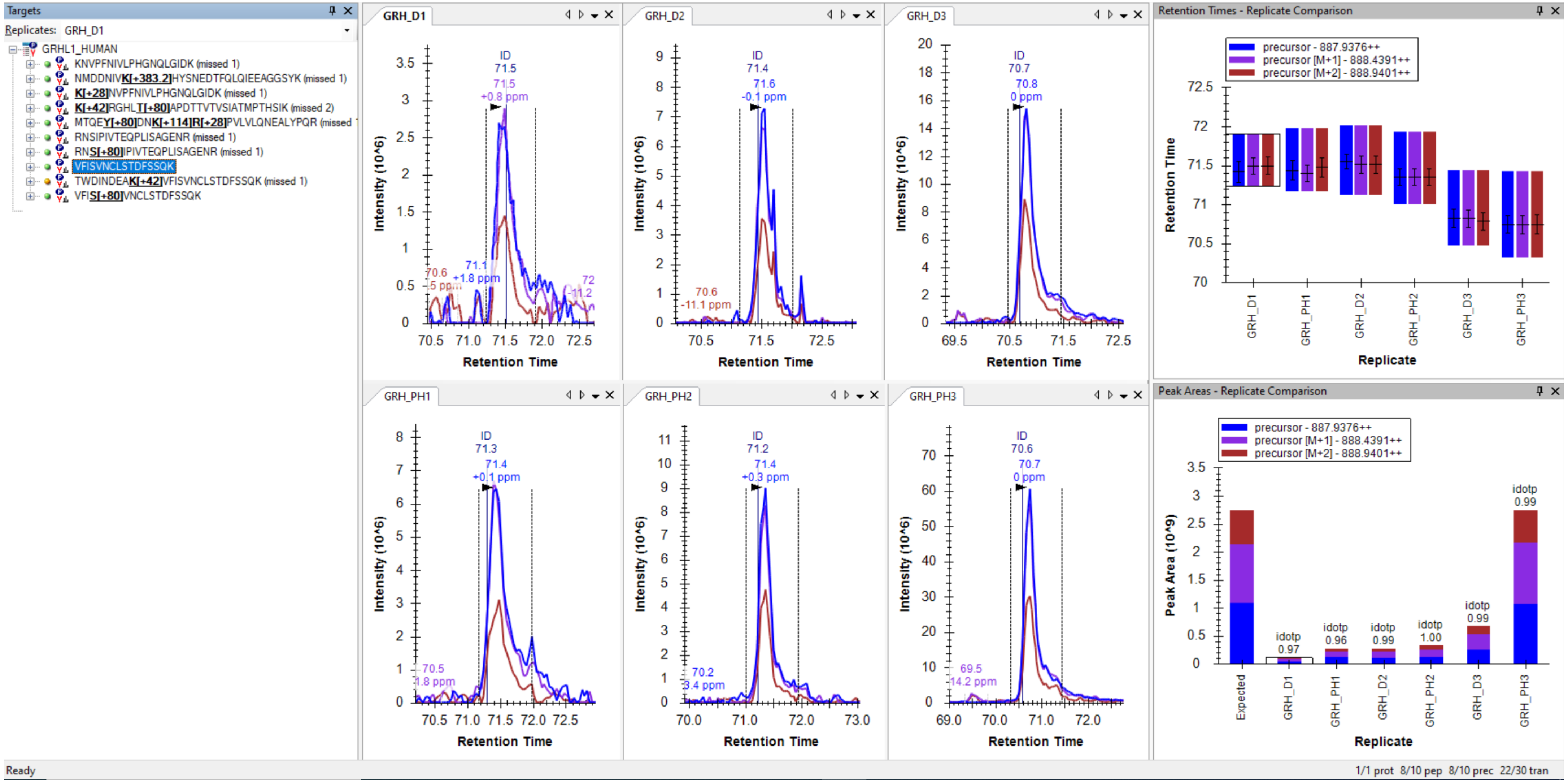

Supplementary Data 7. XICs of GRHL1 post-translational modifications associated with papaverine treatment.

S374 - VFIS**[+80]**VNCLSTDFSSQK
D1-3 = DMSO
PH1-3 = Papaverine

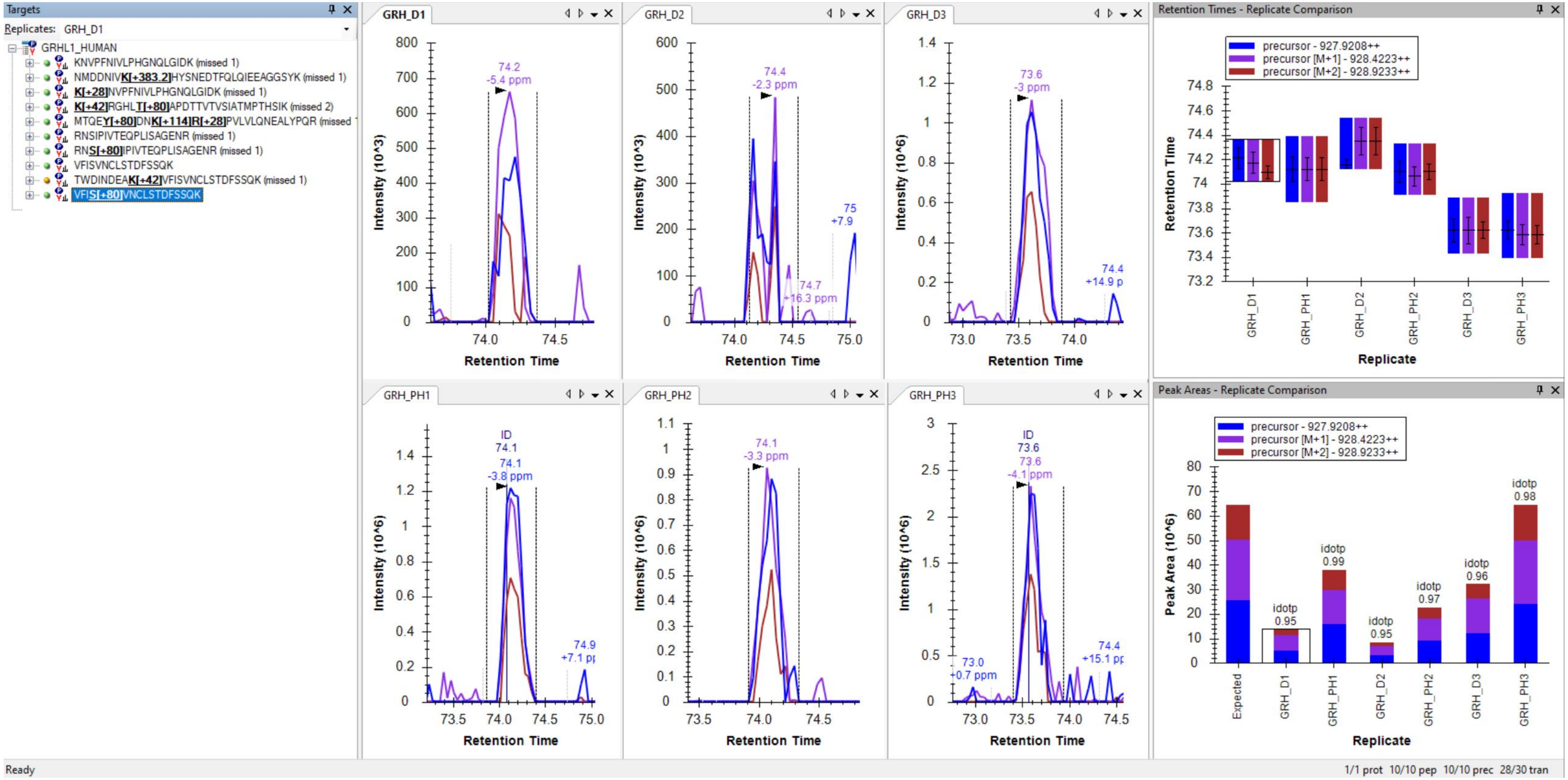

Supplementary Data 7. XICs of GRHL1 post-translational modifications associated with papaverine treatment.

K592 – NMDDNIVK**[+383]**HYSNEDTFQLQIEEAGGSYK
D1-3 = DMSO
PH1-3 = Papaverine

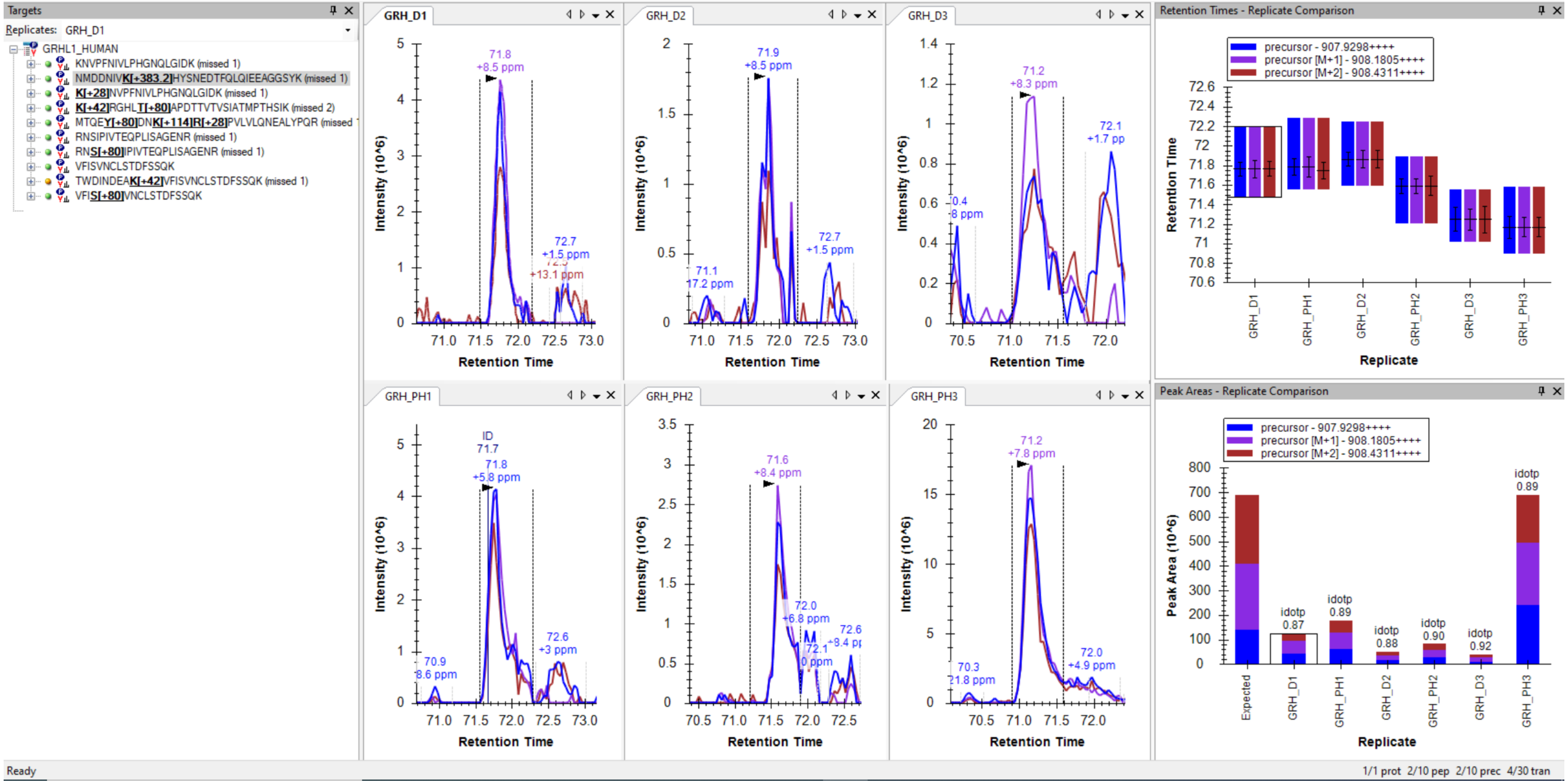

Supplement: Supplementary file 10 — Suppl. Data 7 [file 41467_2021_27732_MOESM10_ESM.pdf]
