# Supplementary material for: Grainyhead 1 acts as a drug-inducible conserved transcriptional regulator linked to insulin signaling and lifespan
Source: Nat Commun. 2022 Jan 10;13:107. doi: 10.1038/s41467-021-27732-4 (PMC8748497; doi:10.1038/s41467-021-27732-4)
Supplement: Supplementary file 11 — Reporting Summary [file 41467_2021_27732_MOESM11_ESM.pdf]

nature portfolio

Corresponding author(s): Michael Ristow

Last updated by author(s): Nov 1, 2021

Reporting Summary

Nature Portfolio wishes to improve the reproducibility of the work that we publish. This form provides structure for consistency and transparency in reporting. For further information on Nature Portfolio policies, see our Editorial Policies and the Editorial Policy Checklist.

Statistics

For all statistical analyses, confirm that the following items are present in the figure legend, table legend, main text, or Methods section.

n/a	Confirmed	
☐	☒	The exact sample size (n) for each experimental group/condition, given as a discrete number and unit of measurement
☐	☒	A statement on whether measurements were taken from distinct samples or whether the same sample was measured repeatedly
☐	☒	The statistical test(s) used AND whether they are one- or two-sided<br>*Only common tests should be described solely by name; describe more complex techniques in the Methods section.*
☐	☒	A description of all covariates tested
☐	☒	A description of any assumptions or corrections, such as tests of normality and adjustment for multiple comparisons
☐	☒	A full description of the statistical parameters including central tendency (e.g. means) or other basic estimates (e.g. regression coefficient) AND variation (e.g. standard deviation) or associated estimates of uncertainty (e.g. confidence intervals)
☐	☒	For null hypothesis testing, the test statistic (e.g. F, t, r) with confidence intervals, effect sizes, degrees of freedom and P value noted<br>*Give P values as exact values whenever suitable.*
☒	☐	For Bayesian analysis, information on the choice of priors and Markov chain Monte Carlo settings
☒	☐	For hierarchical and complex designs, identification of the appropriate level for tests and full reporting of outcomes
☐	☒	Estimates of effect sizes (e.g. Cohen's d, Pearson's r), indicating how they were calculated

Our web collection on statistics for biologists contains articles on many of the points above.

Software and code

Policy information about availability of computer code

Data collection	Confocal imaging: Fluoview FV31S-SW Software V2.4<br>Immunoblotting: Image Lab Touch Software V2.4<br>Mass spectrometry: Exactive MS Series Instrument Control Software V2.9, Orbitrap Tribrid MS Series Instrument Control Software V3.1<br>RNA-Seq: HCS Software for HiSeq 2500, 2000, 1500, and 1000 Systems V2.2.68<br>RT-qPCR: QuantStudio Real-Time PCR Software V1.3<br>BXD data: GeneNetwork2 (www.genenetwork.org/)<br>GTEx and FUSION: GTEx (phs000424.v8.p2) and FUSION (phs001048.v2.p1) from dbGaP (approved request #28650: "Population genetics of human aging")
Data analysis	General data analysis: Microsoft Excel 365 and GraphPad Prism V8.2.0<br>C. elegans lifespan assays statistical analysis: JMP Software V9.0<br>Mass spectrometry data analysis: ProteoWizard V3.0, Mascot search engine V2.5.1.3 (Matrix Science), Scaffold V4.10 (Proteome Software), PEAKS 8.5 (PEAKS X, Bioinformatic Solutions), and MaxQuant Software Suite V1.6.3.3<br>Image analysis: (Fiji Is Just) ImageJ V2.1.0/1.53c in JRE V1.8.0_172 (64-bit)<br>RNA-Seq data analysis: RStudio with R V4.0.0 and custom packages (DESeq2, ezRun, GOseq, GOstats, Subread, and TFBSTools all in their version from Bioconductor release 3.11 compatible with R V4.0.0); FIMO algorithm from the MEME Suite Software V5.1.1<br>BXD, FUSION, and GTEx: custom bash and Python V3.7 scripts with libraries pandas V1.1.4, numpy V1.19.5, matplotlib V3.3.4, seaborn V0.11.1, statsmodels V0.11.0, scipy V1.4.1, pybiomart V0.2.0, gseapy V0.10.2.<br>Custom bash and Python scripts developed in this study for analyses of XXX are deposited at GitHub and can be freely accessed [https://github.com/araldi/Grigolon-et-al_2021].

For manuscripts utilizing custom algorithms or software that are central to the research but not yet described in published literature, software must be made available to editors and reviewers. We strongly encourage code deposition in a community repository (e.g. GitHub). See the Nature Portfolio guidelines for submitting code & software for further information.

Data

Policy information about availability of data

All manuscripts must include a data availability statement. This statement should provide the following information, where applicable:

- Accession codes, unique identifiers, or web links for publicly available datasets
- A description of any restrictions on data availability
- For clinical datasets or third party data, please ensure that the statement adheres to our policy

Publicly accessible databases and datasets used in this study are as follows and in the order they are referenced in the Methods section.
RNA-Seq data analysis: C. elegans reference genome (Genome assembly WBcel235 [https://www.ncbi.nlm.nih.gov/assembly/GCF_000002985.6]).
Tissue-specific correlations of human genes with GRHL1: GTEx database (v8) data for tissue-specific gene TPMs (version 2017-06-05_v8_RNASeQCv1.1.9).
GSEA of human genes correlating with GRHL1: Gene set assembly Human_GO_AllPathways_with_GO_iea_March_01_2021_symbol.gmt retrieved from [download.baderlab.org/EM_Genesets/current_release/Human/symbol].
BXD mice data analysis: Murine Grhl1 expression data and phenotypic traits were obtained from GeneNetwork2 [www.genenetwork.org]. Gene expression datasets analysed: EPFLMouseMuscleCDRMAEx1112, EPFLMouseMuscleCDRMA1211, EPFLMouseMuscleHFDRMAEx1112, EPFLMouseMuscleHFDRMA1211, EPFLMouseLiverCDEx0413, EPFLMouseLiverCDRMA0413, EPFLMouseLiverCDRMA0818, EPFLMouseLiverHFDRMA0818, EPFLMouseLiverHFDRMA0413. Phenotypic traits analysed: 21450 (Lifespan); 17661, 17662 (glycemia during oGTT – AUC); 17663, 176634 (insulin during oGTT - AUC); 17603, 17604 (body weight percentage gain 8-28 weeks).
GTEX and FUSION dataset analysis: Common Fund (CF) Genotype-Tissue Expression Project (GTEx) (phs000424.v8.p2) and The Finland-United States Investigation of NIDDM Genetics (FUSION) Tissue Biopsy Study (phs001048.v2.p1) datasets were obtained from dbGaP (request #28650: "Population genetics of human aging").
Mass spectrometry data analysis: UniProtKB/Swiss-Prot Homo sapiens proteome database (taxonomy 9606, release 2018_09 [https://ftp.uniprot.org/pub/databases/uniprot/previous_major_releases/release-2018_09/knowledgebase]).
The RNA-Seq data generated in this study have been deposited in NCBI's Gene Expression Omnibus (GEO) database under GEO Series accession number GSE159077 [https://www.ncbi.nlm.nih.gov/geo/query/acc.cgi?acc=GSE159077]. The mass spectrometry proteomics data generated in this study have been deposited to the ProteomeXchange (PX) Consortium via the PRIDE database under dataset identifiers PXD021768 [https://www.ebi.ac.uk/pride/archive/projects/PXD021768] and PXD021808 [https://www.ebi.ac.uk/pride/archive/projects/PXD021808]. All other data supporting the findings of this study are available within this paper, its Supplementary Information, and its Supplementary Files. Source data are provided with this paper.

Field-specific reporting

Please select the one below that is the best fit for your research. If you are not sure, read the appropriate sections before making your selection.

☒ Life sciences ☐ Behavioural & social sciences ☐ Ecological, evolutionary & environmental sciences

For a reference copy of the document with all sections, see nature.com/documents/nr-reporting-summary-flat.pdf

Life sciences study design

All studies must disclose on these points even when the disclosure is negative.

Sample size	Sample sizes were not determined a priori using any specific statistical method but are similar to those reported in previous studies for comparable types of experiments, i.e., were selected according to established scientific standards and to allow appropriate statistical calculations. All C. elegans lifespan and health assays used sample sizes as reported previously (e.g., PMID 17908557, 19488025, 26586186). All transcriptomics and proteomics experiments used three independent biological replicates of each respective strain or cell line and treatment condition, as reported previously for comparable analyses (e.g, PMID 26679294, 32203922) and generally considered as sufficient to detect relevant changes (see, e.g., PMID 16212444, 19222236, 27022035 for discussion thereof). The cell-based luciferase reporter primary high-throughput screening used a single dose and replicate to determine initial hit compounds with a stringent cut-off. All hit compounds studied subsequently were first validated in a three-point dose response reporter assay with two to three independent biological replicates, and in each case successfully confirmed to be activators of the GRHL luciferase reporter. All additional cell-based luciferase reporter assays used at least four independent biological replicates. Note that sample sizes and applied statistical tests are reported in full in the manuscript wherever applicable.
Data exclusions	No data were per se excluded from any of the analyses. However, certain censoring criteria apply to C. elegans lifespan assays: Nematodes that crawled off the plates, displayed internal hatching, or a protruding vulva were censored. These exclusion criteria were pre-established and reflect the widely accepted standard for agar-based solid culture C. elegans lifespan assays (PMID 17908557, 28241407).
Replication	All reported data in the manuscript were obtained from independent biological replicates and/or independently repeated experiments (usually at least three replicates), and full outcomes are as reported in the manuscript, its Supplementary Information, and its Supplementary Files. For C. elegans lifespan assays, representative outcomes of individual experiments are depicted in the respective figures and detailed data and statistical analyses of all lifespan assays, including independent repeats, are reported in full in the Supplementary Files. All replicates of non-lifespan experiments are depicted in the respective graphs and sample sizes are as reported in the figure legends. All attempts at replication were successful.
Randomization	Samples/organisms were allocated randomly into the respective experimental groups.
Blinding	The individual main investigators were usually not blinded to group allocations during experimental setup and data analysis, since genotypes (e.g. of C. elegans strains or human cell lines) and treatments (e.g. with a specific RNAi and/or drug compound) had to be carefully confirmed and documented to prevent mistakes. Wherever possible, experiments and readouts after initial setup were performed by technicians or

other investigators in the lab that where unaware of the experimental design, thus excluding bias.

Reporting for specific materials, systems and methods

We require information from authors about some types of materials, experimental systems and methods used in many studies. Here, indicate whether each material, system or method listed is relevant to your study. If you are not sure if a list item applies to your research, read the appropriate section before selecting a response.

Materials & experimental systems

n/a	Involved in the study
☐	☒ Antibodies
☐	☒ Eukaryotic cell lines
☒	☐ Palaeontology and archaeology
☐	☒ Animals and other organisms
☒	☐ Human research participants
☒	☐ Clinical data
☒	☐ Dual use research of concern

Methods

n/a	Involved in the study
☒	☐ ChIP-seq
☒	☐ Flow cytometry
☒	☐ MRI-based neuroimaging

Antibodies

Antibodies used	Primary antibodies:<br>Anti-α-Tubulin antibody, mouse monoclonal clone DM1A, purified from hybridoma cell culture (Sigma-Aldrich, #T6199); Dilution 1:2000 for WB<br>Monoclonal ANTI-FLAG® M2 antibody produced in mouse (Sigma-Aldrich, #F3165); Dilution 1:1000 for WB, 1:200 for ICC/IF<br>Phospho-p38 MAPK (Thr180/Tyr182) antibody (Cell Signaling, #9211); Dilution 1:1000 for WB<br><br>Secondary antibodies:<br>Alexa Fluor® 488 goat anti-mouse IgG (Thermo Fisher Scientific, #A-21141); Dilution 1:1000 for ICC/IF<br>Anti-rabbit IgG, HRP-linked antibody (Cell Signaling, #7074); Dilution 1:2000 for WB<br>Anti-mouse IgG, HRP-linked antibody (Cell Signaling, #7076); Dilution 1:2000 for WB
Validation	Anti-α-Tubulin antibody, mouse monoclonal clone DM1A, purified from hybridoma cell culture (Sigma-Aldrich, #T6199)<br>Species reactivity: bovine, rat, yeast, human, mouse, chicken, fungi, amphibian<br>Validated for WB with human samples (see, e.g., PMID 22348083) and, according to supplier, used in 1500+ peer-reviewed articles. For additional validation information see supplier's website [https://www.sigmaaldrich.com/product/sigma/t6199].<br><br>Monoclonal ANTI-FLAG® M2 antibody produced in mouse (Sigma-Aldrich, #F3165)<br>Species reactivity: all<br>Validated for WB and ICC/IF with human samples (see, e.g., PMID 26772433 and 27294876) and, according to supplier, used in 5000+ peer-reviewed articles. For additional validation information see supplier's website [https://www.sigmaaldrich.com/product/sigma/f3165].<br><br>Phospho-p38 MAPK (Thr180/Tyr182) antibody (Cell Signaling, #9211)<br>Species reactivity: human, mouse, rat, monkey, flies, pig, yeast<br>Validated for WB with human samples (see, e.g., PMID 28497795) and, according to supplier, used in 1900+ peer-reviewed articles. For additional validation information see supplier's website [https://www.cellsignal.com/products/primary-antibodies/phospho-p38-mapk-thr180-tyr182-antibody/9211].<br><br>Alexa Fluor® 488 goat anti-mouse IgG (Thermo Fisher Scientific, #A-21141)<br>Validated by the supplier and used in 300+ peer-reviewed articles.<br><br>Anti-rabbit IgG, HRP-linked antibody (Cell Signaling, #7074)<br>Validated by the supplier and used in 5600+ peer-reviewed articles.<br><br>Anti-mouse IgG, HRP-linked antibody (Cell Signaling, #7076)<br>Validated by the supplier and used in 3000+ peer-reviewed articles.

Eukaryotic cell lines

Policy information about cell lines

Cell line source(s)	The HEK293 wild-type cell line was obtained from a commercial source (CLS Cell Lines Service GmbH, #300192)<br>All other HEK293 cell lines used in this study were derived from this initial cell line, as described in the Methods section.
Authentication	The HEK293 wild-type cell line was authenticated by the supplier and confirmed in-house by growth and morphology.<br>All other derived human cell lines used throughout this study were authenticated/genotyped as described in the Methods

	section.
Mycoplasma contamination	The cell lines were not tested for mycoplasma contamination.
Commonly misidentified lines (See ICLAC register)	The cell lines used in this study are not found in the ICLAC register.

Animals and other organisms

Policy information about studies involving animals; ARRIVE guidelines recommended for reporting animal research

Laboratory animals	The following C. elegans strains used for this publication were provided by the Caenorhabditis Genetics Center (CGC, University of Minnesota, USA): Wild-type N2 (Bristol), RB754 aak-2 (ok524), TJ1052 age-1 (hx546), CB1370 daf-2 (e1370), CF1038 daf-16 (mu86), VC2072 grh-1 (gk960), RB1813 piki-1 (ok2346), EU31 skn-1 (zu135). Strains overexpressing grh-1 were generated as detailed in the Methods section. All experiments, unless explicitly stated otherwise, were performed with young adult hermaphrodite nematodes.
Wild animals	This study did not involve wild animals.
Field-collected samples	This study did not involve samples collected from the field.
Ethics oversight	No ethical approval or guidance was required for this study, since no vertebrate animal organisms were used in any of the experiments.

Note that full information on the approval of the study protocol must also be provided in the manuscript.
